# Association of alcohol use with years lived without major chronic diseases: A multicohort study from the IPD-Work consortium and UK Biobank

**DOI:** 10.1016/j.lanepe.2022.100417

**Published:** 2022-05-29

**Authors:** Solja T. Nyberg, G David Batty, Jaana Pentti, Ida E H Madsen, Lars Alfredsson, Jakob B. Bjorner, Marianne Borritz, Hermann Burr, Jenni Ervasti, Marcel Goldberg, Markus Jokela, Anders Knutsson, Aki Koskinen, Tea Lallukka, Joni V. Lindbohm, Martin L. Nielsen, Tuula Oksanen, Jan H. Pejtersen, Olli Pietiläinen, Ossi Rahkonen, Reiner Rugulies, Martin J. Shipley, Pyry N. Sipilä, Jeppe K. Sørensen, Sari Stenholm, Sakari Suominen, Ari Väänänen, Jussi Vahtera, Marianna Virtanen, Hugo Westerlund, Marie Zins, Archana Singh-Manoux, Mika Kivimäki

**Affiliations:** a Clinicum, Department of Public Health, Faculty of Medicine, University of Helsinki, Tukholmankatu 8B, FI-00014 Helsingin yliopisto, Helsinki, Finland; b Faculty of Social Sciences, University of Tampere, Tampere, Finland; c Finnish Institute of Occupational Health, Helsinki, Finland; d Department of Epidemiology and Public Health, University College London, London, UK; e Department of Public Health, University of Turku and Turku University Hospital, Turku, Finland; f Centre for Population Health Research, University of Turku and Turku University Hospital, Turku, Finland; g National Research Centre for the Working Environment, Copenhagen, Denmark; h Institute of Environmental Medicine, Karolinska Institutet, Stockholm, Sweden; i Centre for Occupational and Environmental Medicine, Stockholm County Council, Sweden; j Bispebjerg University Hospital, Copenhagen, Denmark; k Federal Institute for Occupational Safety and Health (BAuA), Berlin, Germany; l Paris Descartes University, Paris, France; m Inserm UMS 011, Population-Based Epidemiological Cohorts Unit, Villejuif, France; n Department of Psychology and Logopedics, Faculty of Medicine, University of Helsinki, Helsinki, Finland; o Department of Health Sciences, Mid Sweden University, Sundsvall, Sweden; p AS3 Companies, Viby J, Denmark; q Institute of Public Health and Clinical Nutrition, University of Eastern Finland, Kuopio, Finland; r VIVE-The Danish Center for Social Science Research, Copenhagen, Denmark; s Department of Public Health and Department of Psychology, University of Copenhagen, Copenhagen, Denmark; t School of Health Science, University of Skövde, Skövde, Sweden; u School of Educational Sciences and Psychology, University of Eastern Finland, Joensuu, Finland; v Department of Clinical Neuroscience, Karolinska Institutet, Stockholm, Sweden; w Stress Research Institute, Stockholm University, Stockholm, Sweden; x Inserm U1153, Epidemiology of Ageing and Neurodegenerative Diseases, Université de Paris, France

**Keywords:** Alcohol consumption, Binge drinking, Disease-free life-years, Chronic diseases

## Abstract

**Background:**

Heavy alcohol consumption increases the risk of several chronic diseases. In this multicohort study, we estimated the number of life-years without major chronic diseases according to different characteristics of alcohol use.

**Methods:**

In primary analysis, we pooled individual-level data from up to 129,942 adults across 12 cohort studies with baseline data collection on alcohol consumption, drinking patterns, and history between 1986 and 2005 (the IPD-Work Consortium). Self-reported alcohol consumption was categorised according to UK guidelines – non-drinking (never or former drinkers); moderate consumption (1–14 units); heavy consumption (>14 units per week). We further subdivided moderate and heavy drinkers by binge drinking pattern (alcohol-induced loss of consciousness). In addition, we assessed problem drinking using linked data on hospitalisations due to alcohol abuse or poisoning. Follow-up for chronic diseases for all participants included incident type 2 diabetes, coronary heart disease, stroke, cancer, and respiratory disease (asthma and chronic obstructive pulmonary disease) as ascertained via linkage to national morbidity and mortality registries, repeated medical examinations, and/or self-report. We estimated years lived without any of these diseases between 40 and 75 years of age according to sex and characteristics of alcohol use. We repeated the main analyses using data from 427,621 participants in the UK Biobank cohort study.

**Findings:**

During 1·73 million person-years at risk, 22,676 participants in IPD-Work cohorts developed at least one chronic condition. From age 40 to 75 years, never-drinkers [men: 29·3 (95%CI 27·9–30·8) years, women 29·8 (29·2–30·4) years)] and moderate drinkers with no binge drinking habit [men 28·7 (28·4–29·0) years, women 29·6 (29·4–29·7) years] had the longest disease-free life span. A much shorter disease-free life span was apparent in participants who experienced alcohol poisoning [men 23·4 (20·9–26·0) years, women 24·0 (21·4–26·5) years] and those with self-reported heavy overall consumption and binge drinking [men: 26·0 (25·3–26·8), women 27·5 (26·4–28·5) years]. The pattern of results for alcohol poisoning and self-reported alcohol consumption was similar in UK Biobank. In IPD-Work and UK Biobank, differences in disease-free years between self-reported moderate drinkers and heavy drinkers were 1·5 years or less.

**Interpretation:**

Individuals with alcohol poisonings or heavy self-reported overall consumption combined with a binge drinking habit have a marked 3- to 6-year loss in healthy longevity. Differences in disease-free life between categories of self-reported weekly alcohol consumption were smaller.

**Funding:**

Medical Research Council, National Institute on Aging, NordForsk, Academy of Finland, Finnish Work Environment Fund.

**Research in context**

**Evidence before this study**

Both the amount and pattern of alcohol consumption may influence disease-free life-years. We searched for studies examining these associations using PubMed and Embase databases, without language or date restrictions up to June 1, 2021 using the terms: “alcohol”, “drinking pattern”, “binge”, “abuse”, “poisoning”, “healthy years”, “healthy life-years”, “disease-free years”, "disease-free life expectancy", “healthy life expectancy”, and “life expectancy”. We found only three studies on alcohol consumption and disease-free life-years and results were inconsistent: while one study found no difference in disease-free life-years between heavy and moderate drinkers, in another the difference was as much as 5 years. None of the studies examined problem drinking, such as alcohol-related hospitalisations, nor alcohol use patterns, such as binge drinking.

**Added value of this study**

In this prospective, multicohort study of 129,942 men and women from the IPD-Work (Individual Participant Data Meta-analysis in Working Populations) Consortium and 427,621 participants from the UK Biobank study, we used data on self-reported alcohol use, register-based indicators of problem drinking, and years lived without six major chronic diseases: type 2 diabetes, coronary heart disease, stroke, cancer, asthma and chronic obstructive pulmonary disease. In IPD-Work, based on estimations from age 40 to 75 years, participants with hospital-treated alcohol poisoning and heavy alcohol consumers who were also binge drinkers had the lowest average duration of disease-free years. Never drinkers and moderate consumers with no binge drinking habit had 5–6 advantage in disease-free years relative to those with a history of alcohol poisoning, and 2–3 years more compared with heavy consumers with a binge drinking habit. The findings for hospital-treated alcohol poisoning and self-reported alcohol consumption were replicated in the UK Biobank study. In IPD-Work and UK Biobank, differences in disease-free years between self-reported moderate drinkers and heavy drinkers were 1·5 years or less.

**Implications of all the available evidence**

Disease-free life years are an easy-to-understand metric for the lay population complementing information on risk estimates for specific diseases. Evidence on the effects of alcohol use on loss of disease-free life years may therefore add to the development of advice about alcohol use for people. Our data suggest that alcohol abuse, as indicated by alcohol-related poisonings and self-reported heavy consumption combined with binge drinking, are associated with considerable reductions in disease-free life. This loss was comparable to those apparent for obesity, smoking, and physical inactivity.

Alt-text: Unlabelled box

## Introduction

The World Health Organization estimates that harmful alcohol use, including problem drinking, contributes to 1 in 20 deaths and more than 5% of the total burden of disease and injury globally, making it one of the leading risk factors.^1^^,^^2^ Numerous studies have examined the association between overall alcohol consumption and risk of individual chronic diseases, typically reporting the relative risks of these endpoints according to drinking categories. The most replicated findings indicate ‘J’-shaped associations for type 2 diabetes, ischaemic heart disease, stroke and asthma, such that non-drinkers and heavy drinkers experience greater risk.^2^, ^3^, ^4^, ^5^, ^6^ In contrast, there appears to be a linear association between alcohol consumption and cancer risk.^2^

Most studies have used self-report to quantify alcohol use and focused on total alcohol consumption, with less attention being given to the potential deleterious effects of different drinking patterns, such as binge drinking, denoted by the consumption of a large amount of alcohol over a short period of time or the resulting intoxication.^7^^,^^8^ The potential health benefits of moderate drinking also remain unclear as few studies have separated never drinkers from former drinkers who may abstain owing to medical advice. This often leads to an elevated disease risk among non-drinkers that is misattributed to life-long abstinence. In addition, investigators have focused on single disease endpoints rather than the impact on chronic disease in aggregate. However, quantifying the number of disease-free years lost due to all major chronic conditions is important as the net effect on these conditions cannot be obtained from studies on single diseases. Furthermore, absolute metrics, such as disease-free life years complement evidence from relative differences in disease incidence between groups of alcohol users, and thus add to the development of strategies for chronic disease prevention in health policies and clinical guidelines.

We are not aware of any studies on alcohol consumption, drinking patterns and, in non-drinkers, drinking history in relation to healthy life-span – that is, life spent free from major chronic conditions, such as type 2 diabetes, cardiovascular disease, respiratory disease and cancer. To address this limitation, we analysed pooled individual-level data from 12 prospective cohort studies with comprehensive data on characteristics of alcohol use and follow-up for morbidity and mortality. Additionally, we corroborated our main findings in an independent dataset, the UK Biobank study and, for comparison, repeated analyses after replacing our main outcome with disease-free years from alcohol-attributable and partially alcohol-attributable health conditions.^9^

## Methods

### Study population

**Figure 1:**
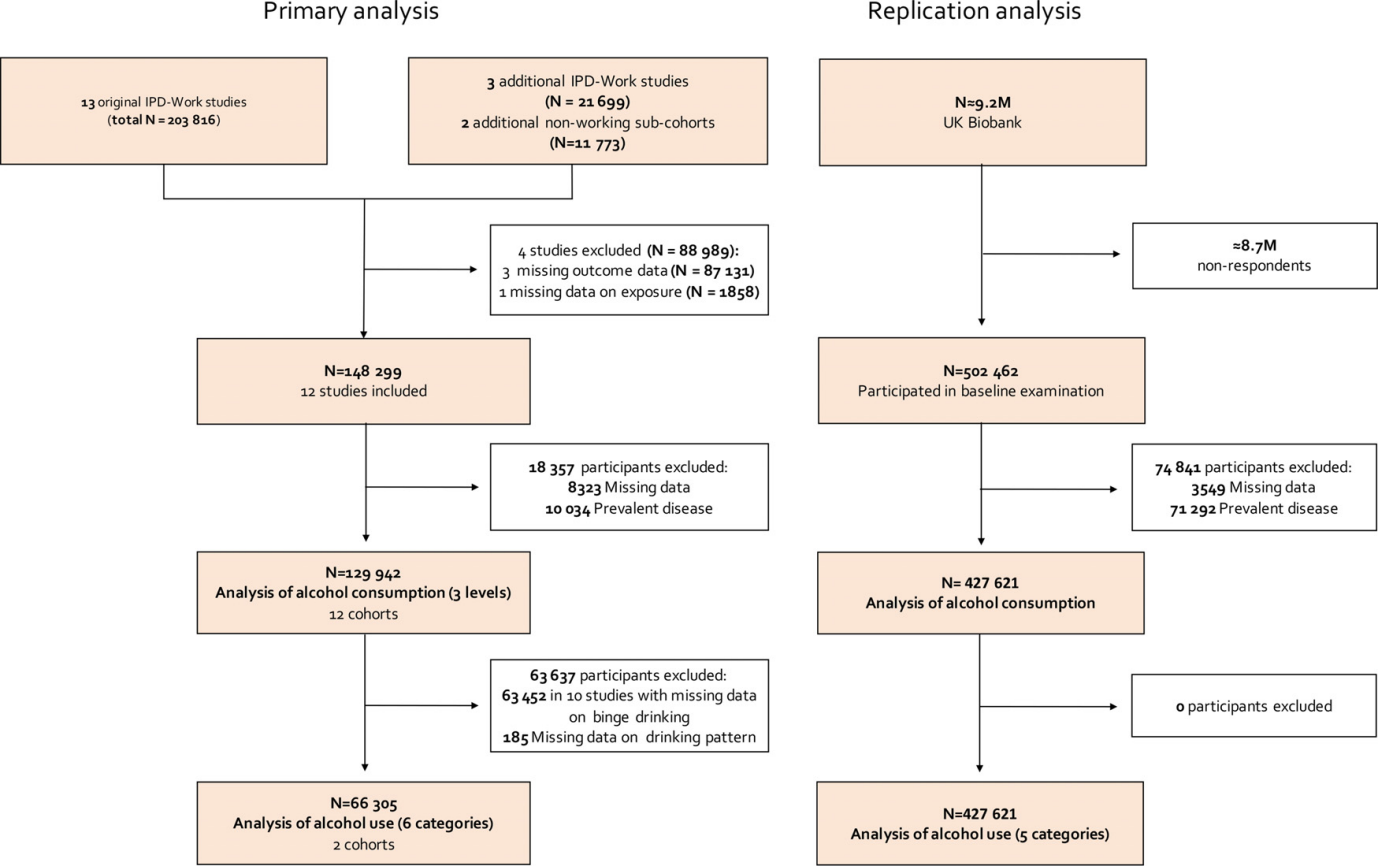
Sample selection for the IPD-Work cohorts (primary analysis) and UK Biobank (replication analysis).

Primary analysis focused on 12 cohort studies featured in the Individual-Participant-Data Meta-Analysis in Working Populations (IPD-Work) consortium:^10^ Whitehall II (United Kingdom); Electricite de France-Gaz de France Employees (GAZEL, France); Copenhagen Psychosocial Questionnaire Study II (COPSOQ-II); Danish Work Environment Cohort Study (DWECS); Intervention Project on Absence and Well-being Study (IPAW, all Denmark); Finnish Public Sector Study (FPS); Health and Social Support (HeSSup); Helsinki Health Study (HHS); and Still Working (all Finland); Work, Lipids, and Fibrinogen Stockholm (WOLF-S); and Work, Lipids, and Fibrinogen Norrland Studies (WOLF-N, all Sweden) (Figure 1). Baseline data collection took place between 1986 and 2005.

These cohort studies were selected because they had data on alcohol consumption at baseline and follow-up for chronic diseases. Participants were included in the analyses if they had baseline data on alcohol consumption and follow-up for six major chronic diseases (129,942 individuals [75,365 women]). In addition, two studies (FPS and HeSSup, *N* = 66,305) had self-reported information on ever drinking and alcohol-induced passing out, an indicator of binge drinking. Details of cohort studies and participants are described in Table 1 and the appendix (pages 2–4, 6)**.**

**Table 1:** Study designs, participants and measures of alcohol use in IPD-Work studies and UK Biobank.

**Study**	**Country**	**Baseline year**	**Population**	**Response rate**	**Number of participants (%) of women**	**Mean (SD) age, years**	**Alcohol surveys**	**Alcohol consumption**		**Alcohol use 6 categories**	Type of follow-up*	**Mean follow-up, years**	**Definition of SES**
								**3-level**	**8-level**				
COPSOQ II	Denmark	2004/5	Occupational	60%	5524 (54)	43.2 (11)	Single item concerning average weekly alcohol consumption with separate responses for number of bottles of beer, glasses of wine or units of liqueur per week. For the present analysis the types of alcohol were summed to obtain weekly alcohol consumption.	·			EHR	5.84	Based on occupational position obtained through linkage to a national register
DWECS 2000	Denmark	2000	General population	76%	7905 (51)	42.0 (13.6)	Daily alcohol consumption was measured using the item: "How much alcohol do you averagely drink per day?” asking the respondent to indicate the number of bottles of beer, glasses of wine, and units of other alcoholic liquors per day. These responses were added and multiplied by 7 to yield weekly alcohol consumption	·			EHR	9.4	Based on occupational position obtained through linkage to a national register
DWECS 2005	Denmark	2005	General population	63%	6007 (53)	40.9 (12.9)	Alcohol was measured using two items regarding average daily alcohol consumption during weekdays and average daily alcohol consumption during the weekend. The number of alcoholic units were multiplied by 5 for weekdays and 2 for weekends and then summed to obtain weekly alcohol consumption.	·			EHR	4.96	Based on occupational position obtained through linkage to a national register
FPS	Finland	2000	Occupational	68%	44527 (81)	44.5 (9.4)	Alcohol consumption was based on the reported amounts of beer, wine or other mild alcoholic beverages and hard liquors. For each category, seven pre-defined answer alternatives were given and weekly consumption was estimated based on the responses. Binge drinking was assessed by requesting the number of occasions the respondent had passed out due to alcohol consumption during the past 12 months. Responses were categorized as 0 vs 1 or more, the latter referring to binge drinking.	·	·	·	EHR	14.5	Occupational title obtained from employers’ register
Gazel	France	1997	Occupational	75%	9501 (27)	50.3 (2.8)	The participant was asked whether or not he consumed wine, beer/cider or aperitifs/digestives during the previous week. For each, the number of days and maximum quantity per day with given response alternatives was asked. Weekly consumption of alcohol was based on the responses.	·	·		Mortality register, repeated questionnaires	12.4	Occupational title obtained from employers’ register
HeSSup	Finland	1998	General population	40%	21963 (59)	36.5 (11.4)	Alcohol consumption was based on the reported amounts of beer, wine or other mild alcoholic beverages and hard liquors. For each category, seven pre-defined answer alternatives were given and weekly consumption was estimated based on the responses. Binge drinking was assessed by requesting the number of occasions the respondent had passed out due to alcohol consumption during the past 12 months. Responses were categorized as 0 vs 1 or more, the latter referring to binge drinking.	·	·	·	EHR	11.5	Participant's self-reported highest educational qualification
HHS	Finland	2000/2	Occupational	67%	6213 (79)	49.3 (6.6)	Alcohol consumption was based on the reported amounts of beer/cider, wine or other mild alcoholic beverages and hard liquors. For each category, seven pre-defined answer alternatives were given and weekly consumption was estimated based on the responses.	·	·		EHR	10.9	Occupational title obtained from employers’ register
IPAW	Denmark	1996/7	Occupational	76%	1915 (67)	40.9 (10.4)	Weekly alcohol consumption was measured using the item “How much alcohol have you drunk on average on a weekly basis during the past year?” asking the respondent to indicate the number of bottles of beer, glasses of wine, and units of other alcoholic liquors per week.	·			EHR	13.09	Based on occupational position obtained through linkage to a national register
Still Working	Finland	1986	Occupational	76%	8813 (23)	40.8 (9.1)	Alcohol consumption was assessed by questions on the number of times the respondent used alcohol per week and whether they drunk "so that they can feel it in their heads" or "so that they can really feel it in their heads".	·			EHR	24.7	Occupational title obtained from employers’ register
Whitehall II	UK	1991/3	Occupational	73%	8000 (31)	49.5 (6.0)	Units of alcohol consumed (spirits, wines, beer) during the last seven days was enquired and weekly consumption was calculated as a sum of the reported amounts.	·	·		EHR, repeated clinical examination, repeated self-report	18.4	Self-reported occupational title
WOLF N	Sweden	1996/8	Occupational	93%	4337 (16)	43.7 (10.3)	The frequency and amount of drinking beer / strong beer / wine / strong wine / spirits was requested and weekly alcohol consumption was derived from the responses.	·	·		EHR	11.2	Self-reported occupational title
WOLF S	Sweden	1992/5	Occupational	76%	5237 (42)	41.2 (10.9)	The frequency and amount of drinking beer / strong beer / wine / strong wine / spirits was requested and weekly alcohol consumption was derived from the responses.	·	·		EHR	13.9	Self-reported occupational title
UK Biobank	UK	2006-2010	General population	5%	427621 (55)	56.5 (8.1)	The frequency and amount of drinking red wine, champagne / white wine, beer / cider, spirits, fortified wine and other (such as alcopops) was requested and weekly alcohol consumption was derived from the responses.	·	·	†	EHR	10.7	Townsend deprivation index at recruitment

*EHR, Electronic Health Records from national registries.

†In UK Biobank only 5 categories.

Replication analysis was based on the UK Biobank study, a large-scale biomedical database (https://www.ukbiobank.ac.uk). Baseline data collection was between 2006 and 2010, and was approved by National Health Service National Research Ethics Service (11/NW/0382). The present analyses of anonymised data did not require further permissions.

IPD-Work cohort studies and UK Biobank have local ethical committee approval.

### Assessment of alcohol use and problem drinking

In IPD-Work cohort studies and UK Biobank, participants were linked to hospitalisations records for alcohol abuse or alcohol poisoning (International Classification of Disease [ICD], version 10 c0des F10.0, F10.1, T51.0, Y15, X45).^11^ Information on alcohol use was extracted from questionnaires completed by participants in all studies (cohort-specific measures of alcohol use are described in the appendix, pages 2–4). Alcohol consumption was based on the total number of units (10 g of ethanol) a participant consumed in a week and categorised according to the UK Chief Medical Officers’ guidelines in which heavy drinking was denoted as a weekly consumption exceeding 14 units for men and women;^12^ moderate drinking was defined as consuming 1–14 units per week. Non-drinkers were divided into self-reported lifelong abstainers and former drinkers. Binge drinking was denoted as a self-report of having passed out at least once during the prior 12 months due to heavy alcohol consumption.^13^

For validation of the self-reported measures of alcohol use, we correlated them with two register-based outcomes: alcohol-related hospitalisations (as described above) and death from alcohol-related causes (ICD-10 codes C01–C06, C09, C10, C12–C15, C22, C32, F10, K70, S00–Y91).^14^ Supporting the validity of self-reported alcohol use in IPD-Work cohort studies and UK Biobank, hazard ratios for death from alcohol-related causes were higher among heavy drinkers compared to moderate drinkers for both men and women (appendix, p. 7-8). Heavy drinkers with binge drinking habit had the highest risks for alcohol-related death in men and women. Similarly, the odds ratio for alcohol-related hospitalisation after baseline was highest among heavy binge drinkers, both in men and women (appendix, p. 9-10). The risk for alcohol-related hospitalisation was elevated in ex-drinker men, suggesting uptake of heavy alcohol use in this group of non-drinkers, but rates were not raised for women.

### Assessment of covariates

In all cohort studies, standard covariates, such as age, sex, and socioeconomic status (SES) were assessed at baseline (for details, see appendix, page 4).^10^ SES was defined based on occupational title obtained from employers' or other registers (in COPSOQ-II, DWECS, FPS, Gazel, IPAW, and Still Working), participant-completed questionnaires (in HHS, Whitehall II, WOLF-N and WOLF-S) or based on the participant's self-reported highest educational qualification (HeSSup). As previously, the harmonised SES was categorised into low, intermediate and high.^10^^,^^15^

### Ascertainment of major chronic diseases and alcohol-attributable conditions during follow-up

In IPD-Work cohort studies and UK Biobank, participants were prospectively linked to national registers for cancer, hospitalisations, prescription reimbursements, and mortality. In some studies, data from 5-yearly clinical examinations or from annual surveys were also collected (description of the ascertainment of major chronic diseases in each study is provided in the appendix, pages 4-5). Ascertainment of the six chronic conditions (type 2 diabetes, coronary heart disease, stroke, cancer, asthma, COPD) are presented in the Box 1.

We excluded participants without follow-up data and those with a baseline record of any of these six chronic diseases (Figure 1). When individual disease outcomes were examined in our analyses, we excluded study participants with that specific disease at baseline. Those with a record of type 1 diabetes at baseline (E10, ICD-10) or 250 (ICD-9 and ICD-8) were additionally defined as prevalent diabetes cases as people with type 1 diabetes at baseline cannot develop type 2 diabetes during follow-up.

We additionally assessed alcohol-attributable conditions (e.g. alcoholic liver disease, alcoholic gastritis, mental and behavioural disorders due to use of alcohol) and partially alcohol-attributable conditions (e.g. tuberculosis, HIV/AIDS and colon and rectum cancer) as post hoc health outcomes.^9^ The ICD-10 diagnostic codes for the first group of diseases were E24·4, F10, G31·2, G62·1, G72·1, I42·6, K29·2, K70, K85·2, K86·0, O35·4, P04·3, Q86·0, R78·0, T51, X45, X65, Y15, Y90, Y91, Z04·0, Z50·2, Z71·4, Z72·1, Z81·1. The 291 diagnostic codes for partially alcohol-attributable conditions are reported in appendix (p. 11).

### Statistical analysis

We carried out the analyses separately for men and women. Disease-free years were defined as the number of life-years between ages 40 and 75 that an individual was free from a diagnosis of any of the six chronic diseases examined. We used three grouping for alcohol use. First, to capture alcohol consumption, drinking history and drinking pattern in our main analysis, we divided participants into six categories of alcohol use: ‘No consumption, never drinker’; ‘No consumption, former drinker’; ‘Moderate consumption without binge drinking’ (reference category); ‘Moderate consumption without binge drinking’; ‘High consumption without binge drinking’; and ‘High consumption with binge drinking’. Second, as in many previous studies, participants were divided into three categories based on their weekly alcohol consumption: non-drinkers, moderate drinkers (reference category) and heavy drinkers. These analyses were reported to allow comparison with other studies. Third, in order to explore higher resolution for alcohol consumption, we divided alcohol consumption into the following 8 categories by the number of average daily doses: 0, 1, 2, 3, 4, 5, 6, and 7 or more units per day. To ensure sufficient power, this analysis was performed for men and women combined.

The UK Biobank data included information on the 3- and 8-category alcohol consumption but did not include items on passing out (binge drinking) for the alcohol use measure. So we divided participants into the following categories of alcohol use: ‘No consumption, never drinker’; ‘No consumption, former drinker’; ‘Moderate consumption’; and ‘High consumption’. In addition, we were able to utilise an additional category of ‘Occasional drinking’ as the fifth category of the UK Biobank alcohol use measure.

To estimate the association between alcohol use categories and disease-free years, hazard ratios with 95% CI for the first disease were calculated using flexible parametric survival models on the cumulative hazards scale, allowing direct estimation of the conditional cumulative hazard function.^16^ Restricted cubic splines with 0 to 4 internal knots (depending on the cohort) were fitted within these models to estimate the baseline hazard for each alcohol consumption category using age as the timescale.

Disease-free years lost in relation to alcohol use categories compared to the referent (moderate consumption without binge drinking in the 6-category alcohol use measure and moderate consumption in the 3-level categorization of alcohol consumption) and in relation to hospitalisations due to alcohol abuse (yes versus no) were calculated as the difference between the areas under the disease-free survival curves from age 40 to 75 years. Area under the curve was computed via numerical integration with a spline-based method. Disease-free years were estimated conditional on survival to age 40 without any of the six chronic diseases investigated. We chose age 40 as this is typically the age at which health checks, monitoring of specific cancers, and assessment of risk of cardiovascular disease, commences.^17^, ^18^, ^19^ Confidence intervals and p-values for disease-free years were estimated via bootstrapping using 1000 independent replications.

In analysis of IPD-Work cohort studies, we used two-stage meta-analysis to combine the results for the 3-level categorization. The results were first calculated separately for each study (first stage), then the study-specific results were pooled using random effects meta-analysis (second stage). Heterogeneity in cohort-specific estimates was assessed with the I² statistic. Due to small numbers of participants in some of the categories in IPD-Work, the results for the 8-level categorization of alcohol consumption, the 6-category measure of alcohol use, and hospitalisations due to alcohol abuse were calculated using a pooled dataset from the IPD-Work studies (Table 1). These models were additionally adjusted for study. We compared results from the 2-stage meta-analyses to those obtained using 1-stage analysis of pooled data. The comparison indicated small differences between the estimates and overlapping confidence intervals, thus supporting the use of one-stage analysis (Appendix, p. 12).

In a sensitivity analysis, we repeated the main analyses of alcohol use with an alternative categorization for alcohol consumption in which 21-unit threshold denotes to heavy consumption (this threshold is used in some countries) and the unit of alcohol is defined at 8 rather than 10 g of ethanol.^10^ To examine whether the association between alcohol consumption and disease-free life-years was apparent across the socioeconomic continuum, we stratified the analyses by categories of socioeconomic status (high, intermediate, low). To minimise confounding by smoking, we repeated the main analysis after excluding smokers from the analytic sample. To address potential survival bias, we conducted a Fine and Gray competing risk analysis with any chronic condition and death as outcomes.^20^ To study disease-free years by single chronic conditions, we examined the associations of alcohol use separately for disease-free years from type 2 diabetes, coronary heart disease, stroke, cancer, asthma and COPD. In addition, for comparison, we repeated the main analysis using two alternative outcomes in which the six chronic diseases were replaced with alcohol-attributable conditions (the first alternative outcome) and partially alcohol-attributable conditions (the second alternative outcome). For both alternative outcomes, we calculated disease-free years between ages 40 and 75 as in the main analysis.

To evaluate reporting bias, we compared the risk of alcohol-related mortality between responders and non-responders using Cox proportional hazard regression adjusted for age and cohort. The validity of self-reported measures of alcohol consumption was examined by studying age- and cohort-adjusted associations of alcohol consumption (3 categories) and alcohol drinking patterns (6 categories) with subsequent hospitalisations due to alcohol abuse and with death from alcohol related causes at follow-up. In addition, we examined the associations of self-reported measures of alcohol consumption with hospitalisations due to alcohol abuse a maximum of five years before baseline and before and after baseline using age- and cohort-adjusted logistic regression.

Data were analysed using SAS 9·4 for Windows and Stata/MP 15·1 for Mac, packages stpm2, metan, and metareg.^21^^,^^22^ Statistical code is available in Appendix (pp. 19–22).

### Role of the funding source

The funders of the study had no role in study design, data collection, data analysis, data interpretation, or writing of the report. STN, JP, and MK had full access to data from FPS, HeSSup, HHS, Gazel, Still Working, Whitehall II, WOLF N, and WOLF S cohort studies; and IEHM and JKS had full access to data from the COPSOQ II, DWECS 2000 and 2005, and IPAW studies. STN and MK had final responsibility for the decision to submit this manuscript for publication.

## Results

In IPD-Work cohort studies, individual-level data were available for 148,299 participants (Figure 1). We excluded 8323 (6%) people owing to missing information on age, sex, alcohol consumption, or chronic diseases. A further 10,034 (7%) individuals with a history of any of the six described chronic diseases were also omitted. Thus, the total analytic sample comprised 129,942 individuals. Compared to participants who responded to alcohol-related questions, the risk of alcohol-related death was more elevated among women who did not (HR 1·80, 95% CI: 1·03–3·15) but there was no difference for men (HR 1·07, 95% CI: 0·44–2·59). The replication cohort consisted of 502,462 individuals who participated in the baseline examination of the UK Biobank study. We excluded 3549 participants due to missing data and a further 71,292 due to prevalent disease.

As shown in Table 1**,** participants of the IPD-Work cohorts comprised 54,577 (42·0 %) men and 75,365 (58·0 %) women. The replication cohort comprised 192,210 (44·9%) men and 235,411 (55·1%) women. In the primary analysis, mean age at baseline and follow-up times were very similar for men (43·6 years and 13·7 years) and women (43·0 years and 13·1 years), the range in mean follow-up being 4·9–24·7 years between studies. During 745,482 person-years at risk, 11,702 (21·4%) men experienced at least one disease event (incidence 15·7 per 1000 person-years). The corresponding figures for women were 10,974 (14·6%) during 988,152 person-years at risk (incidence 11·1 per 1000 person-years).

**Figure 2:**
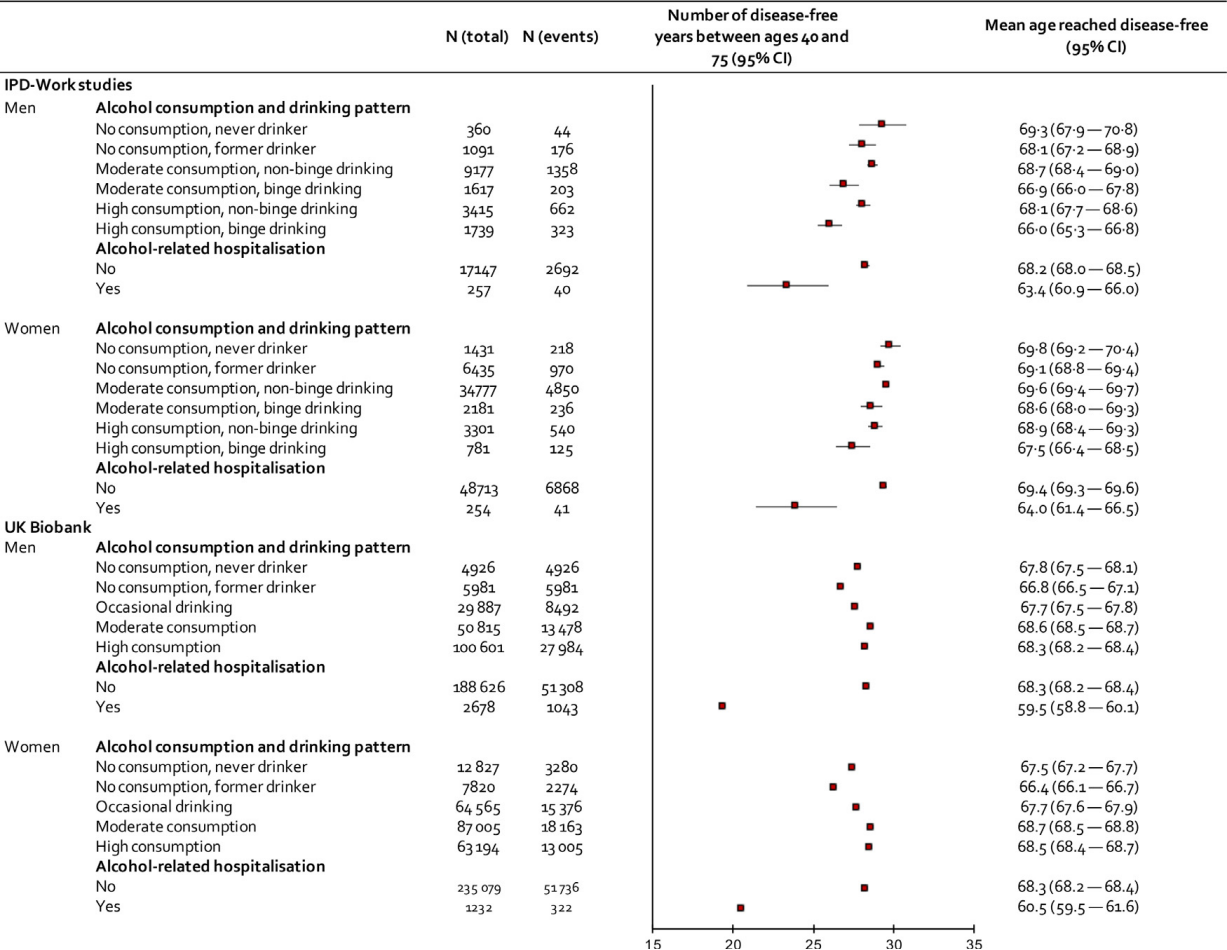
Association of alcohol consumption, binge drinking and alcohol-related hospitalisation with disease-free years between 40 and 75 in IPD-Work cohorts and UK Biobank.

The maximum number of disease-free years between 40 and 75 is 35. As shown in Figure 2 (upper panel), the highest loss of disease-free life-years was found among participants with alcohol-related hospitalisation. Men with alcohol-related hospitalisations had 23·4 (95% 20·9–26·0) years disease-free whereas those without such hospitalisations had 28·2 (95% CI 28·0–28·5). The corresponding figures for women were 24·0 (95% CI 21·4–26·5) and 29·4 (95% CI 29·3–29·6) years. Participants who binge drank irrespective of overall alcohol consumption lost more disease-free years than those who did not report binge drinking. For example, compared to those with moderate consumption and no binge drinking, men and women who consumed alcohol moderately but also reported binge drinking lost 1·75 (*p* < 0·001) and 0·94 (*p* = 0·007) years, respectively. For participants with high alcohol consumption and binge drinking, the loss was 2·65 (men, *p* < 0·001) or 2·09 (women, *p* < 0·001) years of disease-free life.

Compared to participants with moderate consumption without binge drinking, never-drinking was associated with non-significantly longer disease-free life (0·6 years for men, *p* = 0·414 and 0·2 years for women, *p* = 0·471) while for former drinkers it was shorter (-0·6 years for men, *p* = 0·156 and -0·5 years for women, *p* = 0·008) (Figure 2, upper panel). As reported in appendix (p. 13–16), the pattern of results on associations between alcohol use and disease-free years remained essentially the same when we used a higher threshold (>21 units per week) to denote high alcohol consumption, a lower alcohol unit definition (8 grams of ethanol), at each level of socio-economic status, when restricting the analyses to non-smokers only and after addressing competing risk by mortality. In analyses for specific chronic conditions (appendix, p. 17–18), type 2 diabetes and cancer were associated with the lowest estimates for disease-free years regardless of consumption category. With few exceptions, the patterns of the findings were similar, although the associations were slightly weaker between the consumption categories compared to any chronic disease.

**Figure 3:**
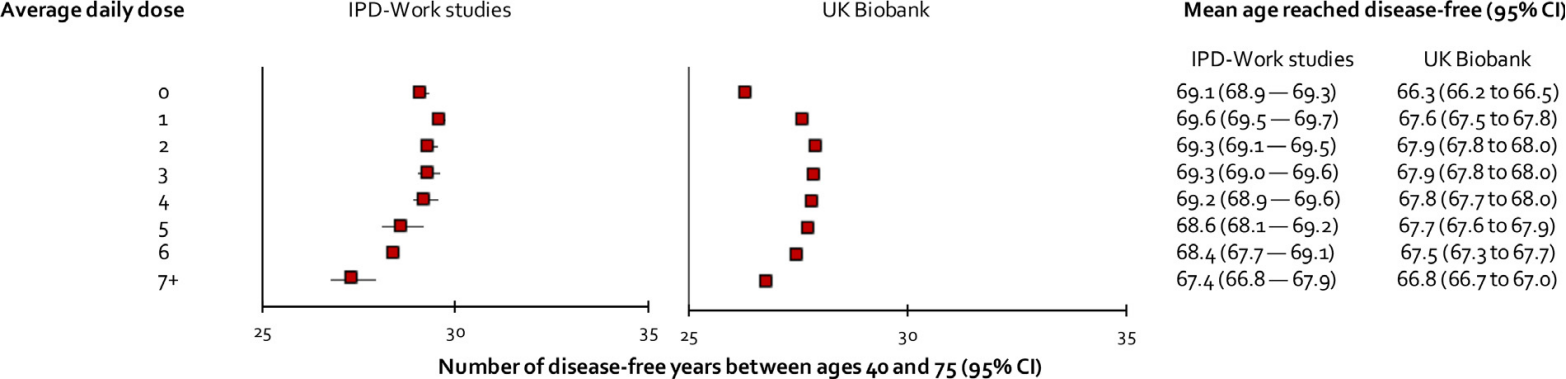
Associations of alcohol consumption defined as daily doses with disease-free years between 40 and 75 in IPD-Work cohorts and UK Biobank.

**Figure 4:**
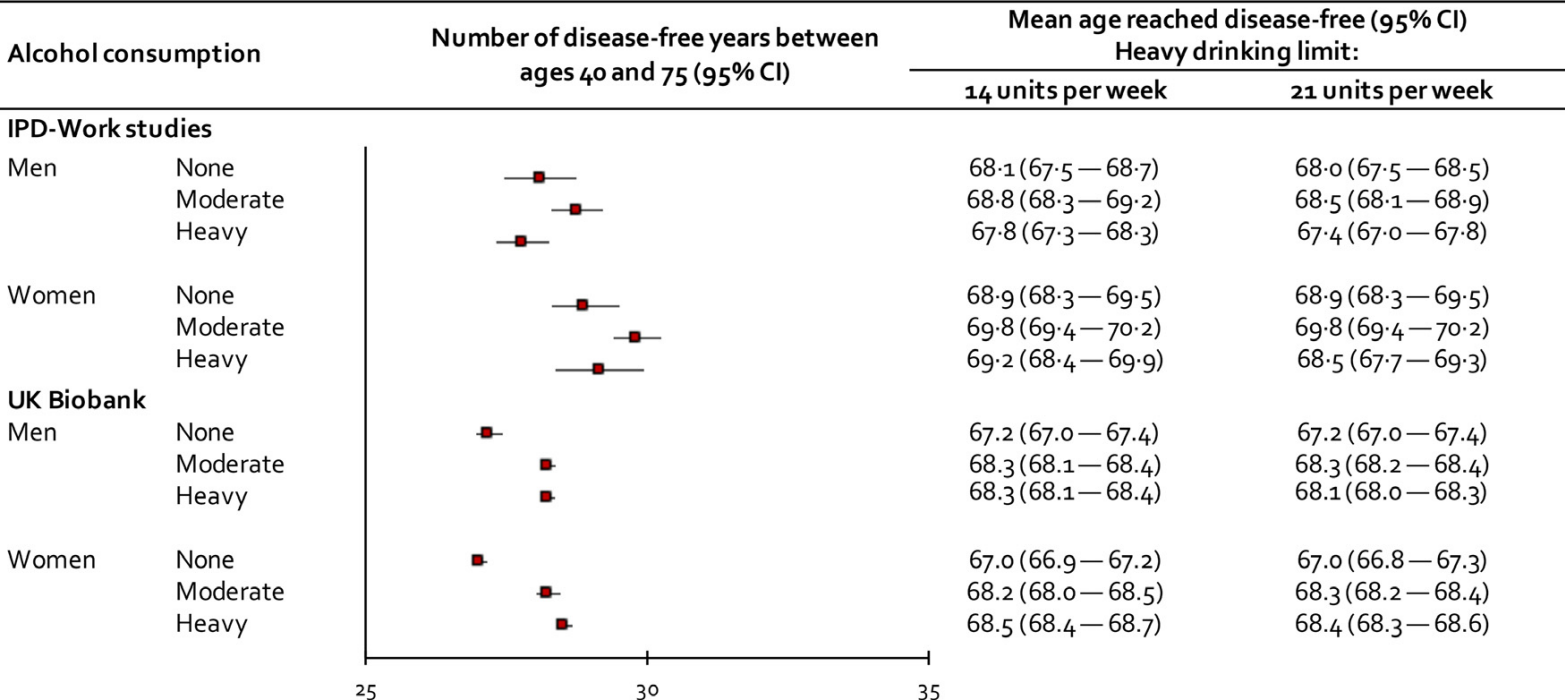
Associations of alcohol consumption with disease-free years between 40 and 75 in IPD-Work cohorts and UK Biobank.

Figure 3 (left panel) shows the association between alcohol consumption defined as daily doses and years of disease-free life between ages 40 and 75. Participants with 0, 2, 3 and 4 daily doses had about equal number of disease-free years (range 29·1 to 29·3) whereas those with 5 daily doses or more had less (27·4, 95%CI 26·8–27·9, to 28·4, 95%CI 27·7–29·1 years) and those with 1 daily dose had slightly more (29·6, 95CI% 29·5–29·7 years). Figure 4 shows the estimated number of disease-free life-years after age 40 using alcohol consumption categorised into 3 groups. As expected, the highest estimates were found for moderate drinkers both in men and women (men: 28·8 years; 95% CI 28·3-29·2; women: 29·8 years; 95% CI 29·4–30·2). Any loss of disease-free life with respect to non-drinking or heavy drinking was small. I^2^ indices ranged between 23·7% (*p* = 0·22) and 46·8% (*p* = 0·04), suggesting that differences in cohort-specific estimates were by chance rather than heterogeneity in effects. In sensitivity analyses in which we used a higher threshold (>21 units per week) to denote heavy alcohol consumption, the pattern of findings remained the same.

Participants in UK Biobank were older than those in IPD-Work (men 56·6 years and women 56·5 years). During a mean follow-up of 10·7 years, 105,400 of them developed at least one incident disease. Replicating findings from IPD-work, history of alcohol-related hospitalisation was associated with an 8- to 9-year loss to disease-free life and the earliest onset of specific diseases and former drinkers lost more disease-free years than never drinkers (Figure 2, lower panel**).** In addition, participants who drank occasionally lost more disease-free years than those with moderate or high consumption. Higher resolution analysis of alcohol consumption showed the longest disease-free life for those consuming 2 rather than 1 daily doses and increased loss of disease-free years began after 6 rather than 5 daily doses (Figure 3, right panel). Surprisingly, the shortest disease-free life was observed among participants who did not consume alcohol. In analysis of 3-level alcohol consumption, non-drinkers had a shorter disease-free life than participants with moderate and high consumption, while little difference in disease-free years were observed between those with moderate and high consumption (Figure 4, lower panel).

Table 2 shows associations of alcohol use with the number of life years free from alcohol-attributable and partially alcohol-attributable diseases between ages 40 and 75. As expected, the number of life years free from alcohol-attributable diseases in never drinkers was near the maximum 35 and reduced in a graded fashion with greater consumption in both IPD-Work cohorts and UK Biobank. Similarly, a lower number of alcohol-attributable disease-free years was observed in participants with compared to without binge drinking habit. Thus, the highest loss of disease-free years was observed in those with high consumption and binge drinking in IPD-Work cohorts (mean number of disease-free years 31·8, 95%CI 31·0–32·6 in men and 32·1, 95%CI 31·1–33·0 in women). As no data from binge drinking were available in UK Biobank, the highest loss of alcohol-attributable disease-free years was found in participants with high consumption (31·9, 95%CI 31·8–32·0 in men and 32·7, 95%CI 32·6–32·8).

**Table 2:** Association of alcohol use with disease-free years from alcohol-attributable and partially alcohol-attributable diseases.

Population	Consumption and drinking pattern	Disease-free years between ages 40 and 75* (95% CI) from	
		Alcohol-attributable dg	Partially alcohol-attributable dg
**IDP-Work studies cohorts**			
Men	No consumption, never drinker	34.7 (34.3–35.2)	28.6 (26.6–30.7)
	No consumption, former drinker	34.6 (34.2–35.0)	28.2 (27.1–29.4)
	Moderate consumption, non-binge drinking	34.5 (34.4–34.7)	29.3 (29.0–29.7)
	Moderate consumption, binge drinking	33.4 (32.7–34.1)	27.0 (25.8–28.2)
	High consumption, non-binge drinking	33.1 (32.7–33.5)	28.8 (28.3–29.3)
	High consumption, binge drinking	31.8 (31.0–32.6)	26.6 (25.6–27.6)
Women	No consumption, never drinker	35.0 (34.9–35.0)	29.4 (28.7–30.1)
	No consumption, former drinker	34.9 (34.8–34.9)	29.1 (28.7–29.5)
	Moderate consumption, non-binge drinking	34.7 (34.7–34.8)	29.5 (29.4–29.6)
	Moderate consumption, binge drinking	33.9 (33.6–34.3)	28.5 (27.7–29.3)
	High consumption, non-binge drinking	34.0 (33.8–34.3)	28.9 (28.4–29.4)
	High consumption, binge drinking	32.1 (31.1–33.0)	27.8 (26.5–29.1)
**UK Biobank**			
Men	No consumption, never drinker	34.8 (34.7–34.9)	26.0 (25.6–26.3)
	No consumption, former drinker	33.5 (33.3–33.7)	25.0 (24.7–25.3)
	Occasional consumption	34.0 (34.0–34.1)	26.6 (26.4–26.7)
	Moderate consumption	33.7 (33.7–33.8)	28.0 (27.9–28.2)
	High consumption	31.9 (31.8–32.0)	27.6 (27.4–27.7)
Women	No consumption, never drinker	34.8 (34.7–34.8)	25.6 (25.4–25.9)
	No consumption, former drinker	34.3 (34.2–34.5)	24.5 (24.2–24.8)
	Occasional consumption	34.2 (34.2–34.3)	26.5 (26.3–26.6)
	Moderate consumption	33.8 (33.8–33.9)	28.0 (27.9–28.1)
	High consumption	32.7 (32.6–32.8)	27.8 (27.7–27.9)

⁎ Maximum number of disease-free years is 35.

**Box 1:** Ascertainment of the chronic conditions from three data sources. EHR, electronic health records.

**Chronic condition**	**ICD-10 code in EHR**	**Clinical examination**	**Self-report**
Type 2 diabetes	E11 from hospitalisation, mortality or drug reimbursement registers	2-h oral glucose tolerance test (fasting glucose ≥7.0 mmol/l, 2 h glucose ≥11.1 mmol/l) or HbA1c >6.5	Self-reported physician-diagnosed diabetes or use of antidiabetic medication
Coronary heart disease	I21–I22 (non-fatal myocardial infarctions) from hospitalisation and I20–I25 (coronary deaths) from mortality registers	Clinical examination using WHO Multinational Monitoring of Trends and Determinants in Cardiovascular Disease (MONICA) Project criteria	Annual self-report questionnaires
Stroke	I60, I61, I63, I64 from hospitalisation or mortality registers		Annual self-report questionnaires
Cancer	C00–C97, hospital discharge, national cancer or mortality registers or the employer's medical register		Annual self-report questionnaires
Asthma exacerbations	J45 or J46 from hospitalisation or mortality registers		Annual self-report questionnaires
COPD exacerbations	J41, J42, J43, and J44 from hospitalisation or mortality registers		

In analysis of partially alcohol-attributable diseases, participants with moderate consumption (in particular those who did not binge drink) had the highest number of disease-free life-years, 29·3 (95%CI 29·0–29·7) in men and 29·5 (95%CI 29·4–29·6) in women of IPD-Work cohorts and 28·0 (95%CI 27·9-28·2) in men and 28·0 (95%CI 27·9-28·1) in women of UK Biobank (Table 2). The greatest loss in disease-free years was observed in participants with high consumption and binge drinking in IPD-Work and among non-drinkers in UK Biobank.

## Discussion

Results from this multicohort study show that between ages 40 and 75, hospitalisation due to alcohol abuse, relative to never drinking and moderate drinking, was associated with 5 to 6 years shorter disease-free lifespan in the IPD-Work cohort studies. The corresponding gap in disease-free life between these groups was 7 to 8 years in the UK Biobank. These replicated findings highlight the strong inverse association between problem drinking and life spent free of major chronic conditions, such as type 2 diabetes, coronary heart disease, stroke, cancer, asthma and chronic obstructive pulmonary disease. Individuals with self-reported heavy overall consumption and a binge drinking habit had a substantial 2- to 3-year loss in healthy longevity. In contrast, differences in disease-free life between self-reported non-drinking, weekly moderate drinking and weekly heavy drinking were smaller, 1·5 years or less.

In comparison with obesity, smoking, and physical inactivity, the role of serious alcohol abuse indicated by alcohol-related poisonings in disease-free years seems comparable, but the role of self-reported high alcohol consumption was more modest. The 6-year loss of disease-free years related to alcohol-related hospitalisations corresponds to the loss for severe obesity compared to normal weight which has been estimated to be 7–9 years^23^^,^^24^ and that for current smoking versus non-smoking which has varied between two and 10 years.^25^, ^26^, ^27^ Physical inactivity has been associated with a 2- to 6-year loss in disease-free life.^25^^,^^27^ When aggregated into a single score, these lifestyle risk factors have yielded up to a 10 years advantage in participants choosing the most favourable combination.^25^^,^^28^ Heavy drinking tends to co-occur with smoking, which is associated with higher loss of disease-free years. We repeated the analysis excluding smokers and the observed association between alcohol use and disease-free life years did not change. This suggests that our findings were not attributable to the effects of smoking.

Our findings on alcohol-related hospitalisations are consistent with a large French study which reported a 12-year shorter life expectancy among patients who have hospitalised due to alcohol use disorders^29^ and findings linking self-reported alcohol use with alcohol-attributable diseases defined in an updated state-of-the-art review based on different dimensions of alcohol use and the burden of disease.^9^ Few studies have examined alcohol consumption patterns in relation to years lived free of most common chronic conditions and we are not aware of other investigations which have also provided disease-specific findings or used non-self-reported measures for alcohol use, such as hospitalisations due to alcohol abuse. A Dutch study of 33,066 individuals, for example, found that, compared with light drinkers, moderate consumers lived 3 months longer without diabetes mellitus, coronary heart disease, stroke, cancer, COPD, asthma, Parkinson's disease, rheumatoid arthritis, osteoarthritis, and inflammatory bowel disease.^30^ In multivariable-adjusted analyses, participants with ‘substantial drinking’ lived 2 months longer free of disease, although this difference disappeared when the group was further divided into heavy and excessive alcohol consumers.^30^ In a study of participants aged 50+ from three European cohorts, abstainers had 1–3 and heavy drinkers 2–5 years fewer life years free of cardiovascular disease than moderate drinkers.^31^ A recent study of over 100,000 participants found little difference in life expectancy free of diabetes, cardiovascular diseases, and cancer between adults with different levels of alcohol consumption.^25^ The variation in the classification of exposure and outcome in these various reports may have contributed to the mixed findings.

Our findings confirmed that non-drinkers are a heterogeneous group.^32^ The observed reduced disease-free life span in former drinkers may be explained by health-related selection such that this group might have been advised to stop drinking because of medical or social problems. However, this group also had elevated rates for clinically verified alcohol-related events after reporting non-drinking indicating that relapses are common among ex-drinkers. This could at least partly explain the ‘J’-shaped curve associations between alcohol consumption and several disease outcomes in the present and previous studies.^2^, ^3^, ^4^, ^5^, ^6^

We also found that never-drinkers had the longest disease-free life span in IPD-Work cohorts, although this was not replicated in the more selected population of UK Biobank participants. The most favourable health span in never-drinkers supports the hypothesis that the net effect of moderate drinking is not beneficial and is consistent with the conclusion drawn by Global Burden of Disease collaboration^2^ and large-scale Mendelian randomisation studies^33^ in that the level of consumption that minimises health loss due to alcohol use is zero.

The association between alcohol use and disease-free life is a net effect of multiple mechanisms that vary between specific chronic diseases. We observed a ‘J’-shaped association of alcohol consumption with diabetes and cardiovascular disease, consistently with previous epidemiological evidence on diabetes mellitus, ischaemic heart disease, heart failure, ischaemic stroke.^9^ Possible cardiometabolic advantages of moderate drinking include a favourable effect of alcohol on lipid levels and insulin sensitivity,^34^^,^^35^ although Mendelian randomization studies using genetic variants as unbiased proxy indicators for alcohol consumption have failed to confirm this hypothesis.^33^^,^^36^ For cancers, we observed a linear association which accords with position statements^37^ classifying alcohol as a carcinogenic beverage that increases the risk of several malignancies in a dose-response fashion and previous reviews of cite-specific cancers which have confirmed a detrimental effect on at least female breast cancer, pancreatic, colorectal, esophageal, laryngeal, liver, oral cavity/pharynx cancers.^9^ Although asthma is not typically considered as alcohol-related condition,^9^ elevated blood acetaldehyde levels have been proposed as the underlying mechanism for alcohol-induced exacerbations of asthma as acetaldehyde leads to degranulation of mast cells or basophils, resulting in a release of histamine, which can induce asthma exacerbation.^38^

The strengths of the present study include the use of a large, harmonised dataset with comprehensive event surveillance via electronic health records, the availability of data that allowed higher resolution stratification by drinking patterns and history than is typically seen in the current literature, and replication of the main findings in a large independent dataset. This study has several limitations. First, coverage of alcohol use in population surveys is low as indicated by comparisons to ‘real consumption’ per capita estimates from multisource modelling studies, such as the World Health Organization's global monitoring system on alcohol.^39^ Such imprecision in measurement of alcohol use and random errors in self-reporting (regression dilution bias) may have contributed to underestimation of the associations of alcohol consumption and binge drinking in the present study, but are an unlikely source of bias for the findings on alcohol-related hospitalisations as those data relied on electronic health records. The validity of the alcohol use measurement in our study was supported by the expected associations of self-reported alcohol use with alcohol-related hospitalisations and deaths from alcohol-related causes (reported in the methods section) as well as the dose-response association of alcohol consumption with alcohol-attributable diseases and their higher incidence among binge drinkers at different levels of consumption (reported in the results section)

Second, methods of assessing alcohol use varied between IPD-Work cohort studies. This is a potential source of error as the way alcohol use is assessed may affect results. However, replication of the main findings in an independent study based on a single large cohort, in sensitivity analyses with alternative operationalisations of alcohol categories and units and in subgroups (men, women, non-smokers, socioeconomic groups) suggests our findings were not significantly affected by heterogeneity. Third, further studies are needed to assess the generalisability of our findings in countries with different drinking cultures, particularly low- and middle-income countries. We found no systematic differences in the associations between alcohol use and disease-free life years in relative terms, but our 3-level measurement of socioeconomic status was crude. Future investigations are warranted to examine effect modification by socioeconomic factors because level of education, individual and family outcomes, and employment status may all have a specific influence on substance use in particular, and on health behaviours and health condition in general.^40^, ^41^, ^42^

Fourth, as this is an observational study, we are unable to determine whether changes in alcohol consumption or drinking habits would affect disease-free years. Without repeat measurement of alcohol intake, it is likely that we have underestimated its impact on disease-free life-expectancy. Lastly, health records, by definition, do not capture undiagnosed conditions and although ascertainment of advanced cancer, stroke, and coronary heart disease (as defined by myocardial infarction and coronary death) is likely to be comprehensive, records of type 2 diabetes, asthma, and COPD in hospital and death registries cover only the most severe cases as these chronic conditions are typically diagnosed and treated in primary care and do not require hospital admissions.

In conclusion, complementing prior evidence on risk estimates for specific diseases our analysis reports estimates of effects of alcohol use on an aggregated endpoint, quantified as loss of disease-free life years, based on multiple large cohort studies. Our data suggest that alcohol abuse, as indicated by alcohol-related poisonings and self-reported heavy consumption combined with binge drinking, is associated with considerable reductions in disease-free life. The shortening of life lived without type 2 diabetes, coronary heart disease, stroke, cancer, asthma and chronic obstructive pulmonary disease was comparable to those apparent for obesity, smoking, and physical inactivity.

## Contributors

All authors participated in designing the study, generating hypotheses, interpreting the data, and critically reviewing the paper. STN and MK, with GDB, wrote the report. STN, IEHM and JKS, with help from JP, supervised data analysis. STN, JP, and MK had full access to data from FPS, HeSSup, HHS, Gazel, Whitehall II, WOLF N, and WOLF S cohort studies; IEHM and JKS had full access to data from the COPSOQ II, DWECS 2000 and 2005, and IPAW studies; and MK had access to UK Biobank dataset. The corresponding author attests that all listed authors meet authorship criteria and that no others meeting the criteria have been omitted.

## Declaration of interests

No disclosures were reported.

## Appendix Supplementary materials

Image, application 1

Image, application 2
