# Supplementary material for: Association of alcohol use with years lived without major chronic diseases: A multicohort study from the IPD-Work consortium and UK Biobank
Source: Lancet Reg Health Eur. 2022 May 29;19:100417. doi: 10.1016/j.lanepe.2022.100417 (PMC9160494; doi:10.1016/j.lanepe.2022.100417)
Supplement: Supplementary file 1 [file mmc1.docx]

**Supplementary appendix**

**List of contents Page**

Description of IPD-Work cohort studies 2

Assessment of age, sex and socioeconomic status 4

Ascertainment of non-communicable diseases 4

Table S1. Additional baseline characteristics of the participants 6

Table S2. Associations between alcohol consumption, binge drinking, alcohol-related hospitalisation and alcohol-related death in the IPD-Work cohorts 7

Table S3. Associations between alcohol consumption, alcohol-related hospitalisation and alcohol-related

death in the UK Biobank 8

Table S4. Associations of alcohol consumption and binge drinking with alcohol-related

hospitalisation in the IPD-Work cohorts 9

Table S5. Associations of alcohol consumption with alcohol-related hospitalisation

in the UK Biobank 10

Table S6. ICD-10 diagnostic codes for the partially alcohol-attributable conditions 11

Table S7. Comparison of the results on the alcohol consumption-disease-free years association between two datasets 12

Table S8. Comparison of Cox model with any chronic condition as the outcome and Fine and Gray competing risk analysis with any chronic condition and death as outcome 13

Figure S1. Associations of alcohol consumption and binge drinking with disease-free years between

ages 40 and 75 in the IPD-Work cohorts, using different thresholds for high consumption 14

Figure S2. Associations of alcohol consumption and binge drinking with disease-free years

between ages 40 and 75 in the IPD-Work cohorts by SES 15

Figure S3. Associations of alcohol consumption and binge drinking with disease-free years

between ages 40 and 75 in the IPD-Work cohorts among non-smokers 16

Figure S4. Associations of alcohol consumption, binge drinking and alcohol-related hospitalisation with individual chronic conditions in the IPD-Work cohorts 17

Figure S5. Associations of alcohol consumption and alcohol-related hospitalisation with individual chronic conditions in UK Biobank 18

Statistical syntax 19

References 23

**Description of IPD-Work cohort studies**

The IPD-Work cohorts included in this study were the Copenhagen Psychosocial Questionnaire study II, Denmark (COPSOQ-II), the Danish Work Environment Cohort Studies from 2000 and 2005, Denmark (DWECS), the Finnish Public Sector Study, Finland (FPS), a cohort study of Électricité de France-Gaz de France employees, France (Gazel), the Health and Social Support Study, Finland (HeSSup), the Helsinki Health Study, Finland (HHS), the Intervention Project on Absence and Well-being study, Denmark (IPAW), Still Working study, Finland, the Whitehall II Study, the UK (Whitehall II), the Work, Lipids and Fibrinogen Study, Norrland, Sweden (WOLF N), and the Work, Lipids and Fibrinogen Study, Stockholm, Sweden (WOLF S). The UK Biobank study was used in replication analyses.

**Copenhagen Psychosocial Questionnaire version II (COPSOQ II)^1^**

COPSOQ II was carried out in 2004-2005. It included a follow up of respondents from COPSOQ I and also a representative sample of Danish residents aged 20-60 at study baseline. The questionnaire was sent to 8 000 individuals from the random sample and 4 732 individuals responded. With respondents, who were originally invited to COPSOQ-I, the total number of participants with a linkage to register data was 5998. The questionnaire could be completed using the posted questionnaire or via internet. COPSOQ-II was approved by and registered with the Danish Data protection agency (registration number: 2004-54-1493). Data on occupational position were obtained through linkage to a national register (the Employment Classification Module).

Weekly alcohol consumption was measured using a single item concerning average weekly alcohol consumption with separate responses for number of bottles of beer, glasses of wine or units of liqueur per week. For the present analysis the types of alcohol were summed to obtain weekly alcohol consumption.

**Danish Work Environment Cohort Study (DWECS), Denmark^2,3^**

DWECS is a split panel survey of working age Danish people. The cohort was established in 1990, when a simple random sample of men and women, aged 18-59, was drawn from the Danish population register. The participants have been followed up at five-year intervals and data from the years 2000 and 2005 were used for the present analysis. In 2000, 11 437 individuals were invited to participate and 8583 agreed to do so. 8552 of them were successfully linked with register data. In 2005, an additional random sample of 8545 individuals were invited to participate of which 5176 agreed to do so. First time respondents originally invited for DWECS 2000 but who did not respond that time, were additionally included in this sample with a total of 6741 participants successfully linked with registers. In Denmark, questionnaire- and register-based studies do not require ethics committee approval. DWECS was approved by and registered with the Danish Data protection agency (registration number: 2007-54-0059).

For DWECS 2000, daily alcohol consumption was measured using the item: How much alcohol do you averagely drink per day?” asking the respondent to indicate the number of bottles of beer, glasses of wine, and units of other alcoholic liquors per day. These responses were added and multiplied by 7 to yield weekly alcohol consumption.

For DWECS 2005, alcohol was measured using two items regarding average daily alcohol consumption during weekdays and average daily alcohol consumption during the weekend, respectively. The number of alcoholic units were multiplied by 5 for weekdays and 2 for weekends and then summed to obtain weekly alcohol consumption.

**Finnish Public Sector study (FPS), Finland^4^**

The Finnish Public Sector study is a prospective cohort study comprising the entire public sector personnel of 10 towns (municipalities) and 21 hospitals in the same geographical areas. Participants, who were recruited from employers' records in 2000-2002, were individuals who had been employed in the study organisations for at least six months prior to data collection. 48 592 individuals (9 337 men and 39 255 women aged 17 to 65) responded to the questionnaire. Ethical approval was obtained from the ethics committee of the Finnish Institute of Occupational Health.

Alcohol consumption was based on the reported amounts of beer, wine or other mild alcoholic beverages and hard liquors. For each category, seven pre-defined answer alternatives were given and weekly consumption was estimated based on the responses. Binge drinking was assessed by requesting the number of occasions the respondent had passed out due to alcohol consumption during the past 12 months. Responses were categorized as 0 vs 1 or more, the latter referring to binge drinking.

**Gazel, France^5^**

Gazel is a prospective cohort study of 20 625 employees (15 011 men and 5 614 women) of France's national gas and electricity company, Electricité de France-Gaz de France (EDF-GDF). Since the study baseline in 1989, when the participants were aged 35–50 years, they have been posted an annual follow-up questionnaire to collect data on health, lifestyle, individual, familial, social, and occupational factors. Gazel in 1997 was treated as a baseline year for our analyses. 11 448 individuals participated that year. The GAZEL study received approval from the national commission overseeing ethical data collection in France (Commission Nationale Informatique et Liberté).

Regarding alcohol consumption, the participant was asked whether or not he consumed wine, beer/cider or aperitifs/digestives during the previous week. For each, the number of days and maximum quantity per day with given response alternatives was asked. Weekly consumption of alcohol was based on the responses.

**Health and Social Support (HeSSup), Finland^6^**

The Health and Social Support (HeSSup) study is a prospective cohort study of a stratified random sample of the Finnish population in the following four age groups: 20–24, 30–34, 40–44, and 50–54. The participants were identified from the Finnish population register and posted an invitation to participate, along with a baseline questionnaire, in 1998. 25 898 individuals responded to the questionnaire in 1998. The Turku University Central Hospital Ethics Committee approved the study.

Alcohol consumption was based on the reported amounts of beer, wine or other mild alcoholic beverages and hard liquors. For each category, seven pre-defined answer alternatives were given and weekly consumption was estimated based on the responses.

**Helsinki Health Study (HHS), Finland^7^**

The Finnish Helsinki Health Study (HHS) is a prospective cohort study comprising all employees of the City of Helsinki, who turned 40, 45, 50, 55, or 60 years in 2000-2002. We included in this study all participants who responded to the baseline survey (n=8960, response rate 67%, 80% women) and provided an informed written consent to combine their survey responses with retrospective and prospective registerbased follow-up data on different diseases and mortality (n=6603). Ethical approvals for this study were obtained from the ethics committees of the health authorities of the City of Helsinki, and the Department of Public Health, University of Helsinki.

Alcohol consumption was based on the reported amounts of beer/cider, wine or other mild alcoholic beverages and hard liquors. For each category, seven pre-defined answer alternatives were given and weekly consumption was estimated based on the responses.

**Intervention Project on Absence and Well-being (IPAW), Denmark^8^**

IPAW is a 5-year psychosocial work environment intervention study including 22 intervention and 30 control work places in three organisations (a large pharmaceutical company, municipal technical services and municipal nursing homes) in Copenhagen, Denmark. The baseline questionnaire was posted to all the employees at the selected work-sites between 1996 and 1997. Of the 2 721 employees who worked at the 52 IPAW sites, 2 068 men and women completed the baseline questionnaire and 2055 were successfully linked with register data. Interventions took place at 22 workplaces during 1996-98 at the organisational and interpersonal level. IPAW was approved by and registered with the Danish Data Protection Agency (registration number: 2000-54-0066).

Weekly alcohol consumption was measured using the item “How much alcohol have you drunk on average on a weekly basis during the past year?” asking the respondent to indicate the number of bottles of beer, glasses of wine, and units of other alcoholic liquors per week.

**Still Working, Finland^9^**

Still Working is an ongoing prospective cohort study. In 1986, the employees (n = 12 173) at all Finnish centres of operation of Enso Gutzeit (a forestry products manufacturer) were invited to participate in a questionnaire survey on demographic, psychosocial and health-related factors, and 9 282 individuals participated. The study was approved by the ethics committee of the Finnish Institute of Occupational Health.

Alcohol consumption was assessed by questions on the number of times the respondent used alcohol per week and whether the effect of alcohol use led to any symptoms.

**Whitehall II, UK^10^**

The Whitehall II study is a prospective cohort study set up to investigate socioeconomic determinants of health. At study baseline in 1985-1988, 10 308 civil service employees (6 895 men and 3 413 women) aged 35-55 and working in 20 civil service departments in London were invited to participate in the study. The Whitehall II study protocol was approved by the University College London Medical School committee on the ethics of human research. Written informed consent was obtained at each data collection wave.

Units of alcohol consumed (spirits, wines, beer) during the last seven days was enquired and weekly consumption was calculated as a sum of the reported amounts.

**WOLF (Work, Lipids, and Fibrinogen) Stockholm and WOLF Norrland studies, Sweden^11,12^**

The WOLF (Work, Lipids, and Fibrinogen) Stockholm study is a prospective cohort study of 5 698 people (3 239 men and 2 459 women) aged 19–70 and working in companies in Stockholm county. WOLF Norrland is a prospective cohort of 4 718 participants aged 19-65 working in companies in Jämtland and Västernorrland counties. At study baseline the participants underwent a clinical examination and completed a set of health questionnaires. For WOLF Stockholm, the baseline assessment was undertaken at 20 occupational health units between November 1992 and June 1995 and for WOLF Norrland at 13 occupational health service units in 1996-98. The Regional Research Ethics Board in Stockholm, and the ethics committee at Karolinska Institutet, Stockholm, Sweden approved the study.

The frequency and amount of drinking beer / strong beer / wine / strong wine / spirits was requested and weekly alcohol consumption was derived from the responses.

**UK Biobank**

Uk Biobank is an ongoing prospective cohort study. Approximately 9.2 million invitations were mailed and during 2006-2010, over half a million men and women aged 40-69 years from the United Kingdom participated. The baseline data collection involved questionnaire and physical measurements. Our analysis was done under a generic approval from the National Health Service National Research Ethics Service (11/NW/0382).

The frequency and amount of drinking red wine, champagne / white wine, beer / cider, spirits, fortified wine and other (such as alcopops) was requested and weekly alcohol consumption was derived from the responses.^13^

## **Assessment of age, sex and socioeconomic status**

## Information on sex and age was obtained from population registries or interview (COPSOQ II, DWECS 2000 and 2005, FPS, Gazel, HHS, IPAW, Still Working, WOLF N, WOLF S) or from questionnaires completed by participants (HeSSup, Whitehall II). SES was based on occupational title obtained from employers’ or other registers or questionnaires completed by participants and categorised into low, intermediate, or high. In HeSSup, SES was based on the participant’s self-reported highest educational qualification. Participants who were self-employed or who had missing data on job title were included in the analyses in the “other” category for SES.

In the UK Biobank, sex and age of the participants were obtained from registries. This information could be amended by the participant upon arrival at the Assessment Centre. SES was based on Townsend deprivation index at recruitment.^14^

## **Ascertainment of major non-communicable diseases during follow-up**

Participants were linked to national registers of hospitalisations, prescription reimbursements and mortality. In Whitehall II, participants additionally attended to 5-yearly clinical examinations. Data from annual surveys during the follow-up were available for the participants of the Gazel study.

Linked records of major chronic diseases covered both baseline and follow-up. The outcome of interest in the present study was the first record of either incident type 2 diabetes, coronary heart disease, stroke, cancer, asthma or COPD. These specific diseases were selected because they are the commonest major non-communicable diseases in developed countries^15,16^ and targets prioritised for global disease prevention by the WHO.

Incident type 2 diabetes was defined as the first record of diagnosis corresponding to ICD-10 code E11. We collected records from hospital admissions and discharge registers and mortality registers with a mention of diagnosis of type 2 diabetes in any of the diagnosis codes. Additionally, in the Finnish datasets (FPS, HeSSup, HHS and Still working), participants were also defined as an incident type 2 diabetes case the first time they appeared in the nationwide drug reimbursement register as eligible for type 2 diabetes medication.^17^ In the Whitehall II study, type 2 diabetes was ascertained by 2-h oral glucose tolerance test administered every 5 years^18^ using World Health Organization criteria and complemented by self-reports of diabetes diagnosis and medication.^19^ In the Gazel study, non-fatal cases were based on self-report from annual questionnaires.

Coronary heart disease events were identified from hospital discharge and mortality registers, annual self-report questionnaires, or clinical screening using WHO Multinational Monitoring of Trends and Determinants in Cardiovascular Disease (MONICA) Project criteria. We included all non-fatal myocardial infarctions that were recorded as I21–I22 (ICD-10) or 410 (ICD-9) and coronary deaths recorded as I20–I25 (ICD-10) and 410–414 (ICD-9) in any of the diagnose codes.

Incident stroke was defined with hospital and mortality records (I60, I61, I63, I64 in ICD-10; 430, 431, 433, 434, 436 in ICD-9).^20,21^ In the Gazel study, non-fatal stroke cases were based on self-report from annual questionnaires.

Cancers, C00–C97 (ICD-10 any cancer), were identified via national cancer, hospital or mortality records, except for Gazel, in which incident cancer events were ascertained from the employer’s medical register and by confirming any self-reported cancer diagnosis with the participant’s physician.^22^

Severe asthma (J45 or J46 in ICD-10 or 493 in ICD-9) and COPD exacerbations (J41, J42, J43, and J44 in ICD-10, or 491, 492, and 496 in ICD-9) were ascertained from hospital discharge and death registers in all studies except for Gazel, in which non-fatal asthma events were based on self-report from annual questionnaires and non-fatal COPD was not available.^23,24^

Participants with missing data on these outcomes and those with a record of these diseases already at baseline were excluded from the analyses. We also excluded participants with a record of type 1 diabetes at baseline: E10 (ICD-10) or 250 (ICD-9 and ICD-8).^25^

In UK Biobank, study participants were linked to the UK National Health Service’s Hospital Episode Statistics (HES) database for hospital admissions and the NHS Central Registry for mortality from 18-Mar-1995 to 31-Mar-2021.

**Table S1. Additional baseline characteristics of the participants**

**
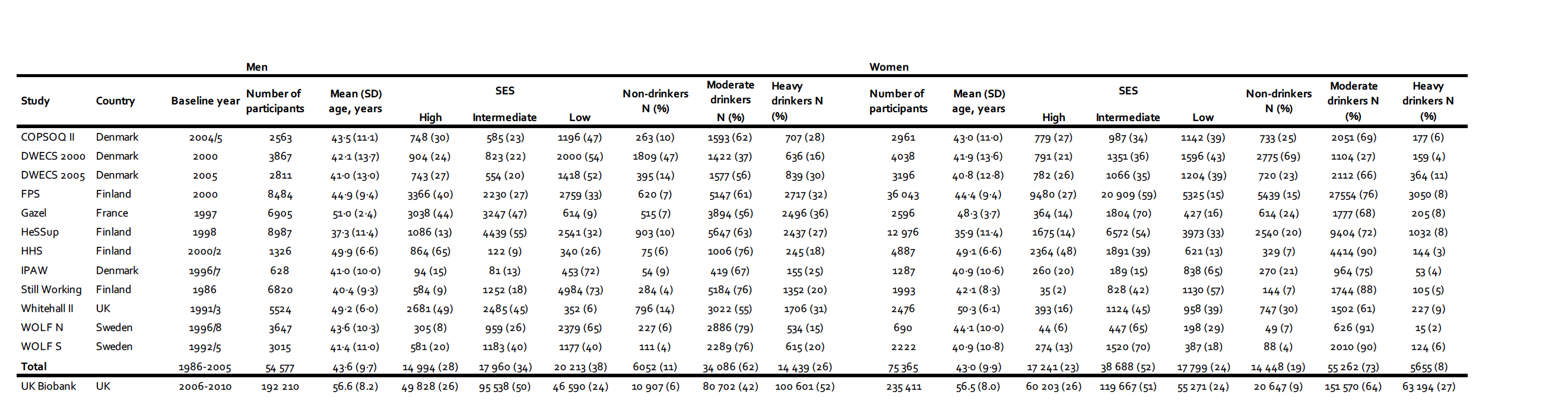
**

**Table S2. Associations between alcohol consumption, binge drinking, alcohol-related hospitalisation and alcohol-related death in the IPD-Work cohorts**

**Table S3. Associations between alcohol consumption, alcohol-related hospitalisation and alcohol-related death in the UK Biobank**

**Table S4. Associations of alcohol consumption and binge drinking with alcohol-related hospitalisation in the IPD-Work cohorts**

**Table S5. Associations of alcohol consumption with alcohol-related hospitalisation in the UK Biobank**

**Table S6.** ICD-10 diagnostic codes for the partially alcohol-attributable conditions

| **Infectious diseases:** | |  |  |  |  |  |
| --- | --- | --- | --- | --- | --- | --- |
| Tuberculosis, HIV/AIDS and other sexually transmitted diseases, | | | | |  |  |
| lower respiratory infections: pneumonia | | |  |  |  |  |
| A10-A14, A15–A19.9, B90–B90.9, K67.3, K93.0, M49.0, P37.0, B20-B24.9 | | | | | |  |
| A50–A58, A60–A60.9, A63–A63.8, B63, I98.0, K67.0–K67.2, M03.1, M73.0–M73.1 | | | | | |  |
| N70–N71.9, N73–N74.8, A48.1, A70, J09–J15.8, J16–J16.9, J20–J21.9, P23.0–P23.4 | | | | | |  |
| **Cancers:** |  |  |  |  |  |  |
| Lip and oral cavity cancer, nasopharynx cancer, other pharynx cancer, oesophagus cancer, | | | | | |  |
| stomach cancer, colon and rectum cancer, liver cancer, pancreatic cancer, larynx cancer, | | | | | |  |
| trachea, bronchus and lung cancer, female breast cancer | | | |  |  |  |
| C0–C08.9, D00.00–D00.07, D10.0–D10.5, D11–D11.9, D37.01–D37.04, D37.09 | | | | | |  |
| C11–C11.9, D00.08, D10.6, D37.05, C09–C10.9, C12–C13.9, D10.7 | | | | |  |  |
| C15–C15.9, D00.1, D13.0, C16–C16.9, D00.2, D13.1, D37.1 | | | | |  |  |
| C18–C21.9, D01.0-D01.3, D12-D12.9, D37.3–D37.5, C22–C22.9, D13.4 | | | | |  |  |
| C25–C25.9, D13.6–D13.7, C32–C32.9, D02.0, D14.1, D38.0 | | | | |  |  |
| C33–C34.92, D02.1–D02.3, D14.2–D14.32, D38.1 | | | |  |  |  |
| C50–C50.929, D05–D05.92, D24–D24.9, D48.6–D48.62, D49.3, N60–N60.99 | | | | | |  |
| **Diabetes mellitus:** | |  |  |  |  |  |
| E10–E10.11, E10.3–E11.1, E11.3–E12.1, E12.3–E13.11, E13.3–E14.1, E14.3–E14.9, | | | | | |  |
| P70.0–P70.2, R73–R73.9 | |  |  |  |  |  |
| **Neuropsychiatric disorders:** | |  |  |  |  |  |
| Alzheimer’s disease and other dementias, major depressive disorder, | | | | |  |  |
| epilepsy / epilepsy impairment envelope | | |  |  |  |  |
| F00–F03.91, G30–G31.1, G31.8–G31.9, F32, F33, G40–G41.9 | | | | |  |  |
| **Cardiovascular diseases:** | |  |  |  |  |  |
| Hypertensive heart disease, ischaemic heart disease, cardiomyopathy, atrial fibrillation and flutter, | | | | | | |
| heart failure, ischaemic stroke, haemorrhagic and other non-ischaemic stroke, oesophageal varices | | | | | | |
| I11–I11.9, I20–I25.9, A39.52, B33.2–B33.24, D86.85, I40–I43.9, I51.4–I51.5 | | | | | |  |
| I48–I48.92, I50, I11.0, I13.0, I13.2, G45–G46.8, I63–I63.9, | | | | |  |  |
| I65–I66.9, I67.2–I67.3, I67.5–I67.6, I69.3–I69.398, I60–I61.9, I62.0–I62.03 | | | | | |  |
| I67.0–I67.1, I68.1–I68.2, I69.0–I69.298, I85 | | | |  |  |  |
| **Gastrointestinal diseases:** | |  |  |  |  |  |
| Cirrhosis of the liver | |  |  |  |  |  |
| B18–B18.9, I85–I85.9, I98.2, K70–K70.9, K71.3–K71.51, K71.7 | | | | |  |  |
| K72.1–K74.69, K74.9, K75.8–K76.0, K76.6–K76.7, K76.9 | | | | |  |  |

**Table S7. Comparison of the results on the alcohol consumption-disease-free years association between two datasets**

|  |  |  |
| --- | --- | --- |
| **Sex** | **Mean age reached disease-free (95% CI)** | |
| **Alcohol consumption** | **All cohorts*** | **Pooled dataset*** |
| **Men** |  |  |
| None | 68.1 (67.5 — 68.7) | 68.4 (67.7 — 69.1) |
| Moderate | 68.8 (68.3 — 69.2) | 68.5 (68.2 — 68.8) |
| Heavy | 67.8 (67.3 — 68.3) | 67.6 (67.2 — 68.0) |
| **Women** |  |  |
| None | 68.9 (68.3 — 69.5) | 69.2 (69.0 — 69.5) |
| Moderate | 69.8 (69.4 — 70.2) | 69.5 (69.4 — 69.7) |
| Heavy | 69.2 (68.4 — 69.9) | 68.6 (68.2 — 69.1) |

*Result from all cohorts were from a two-stage meta-analysis model using maximum data.

Pooled dataset included the FPS and HeSSup studies. The latter model was adjusted for study.

**Table S8. Comparison of Cox model with any chronic condition as the outcome and Fine and Gray competing risk analysis with any chronic condition and death as outcomes**


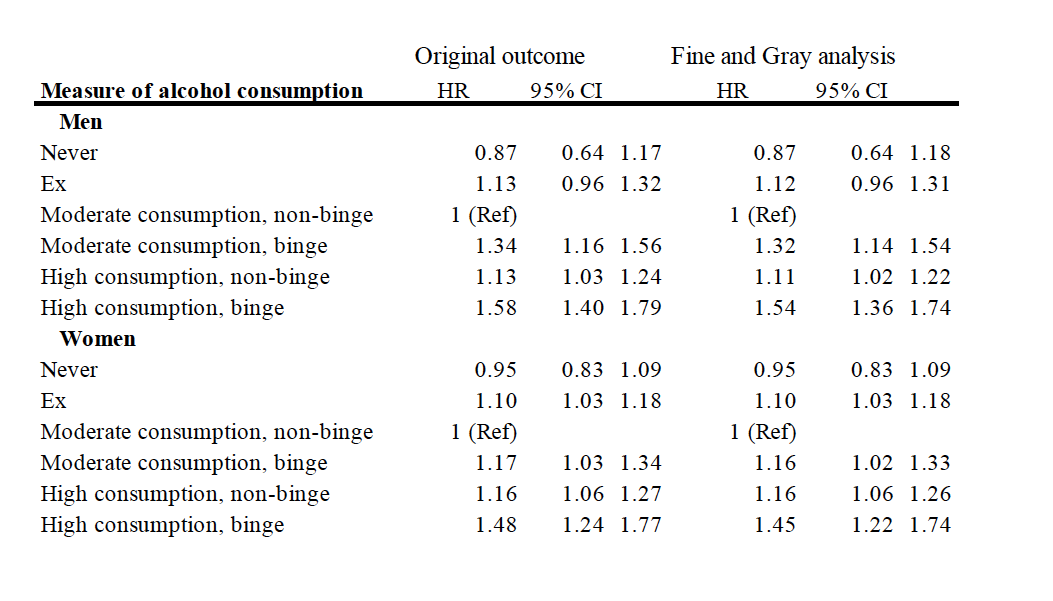


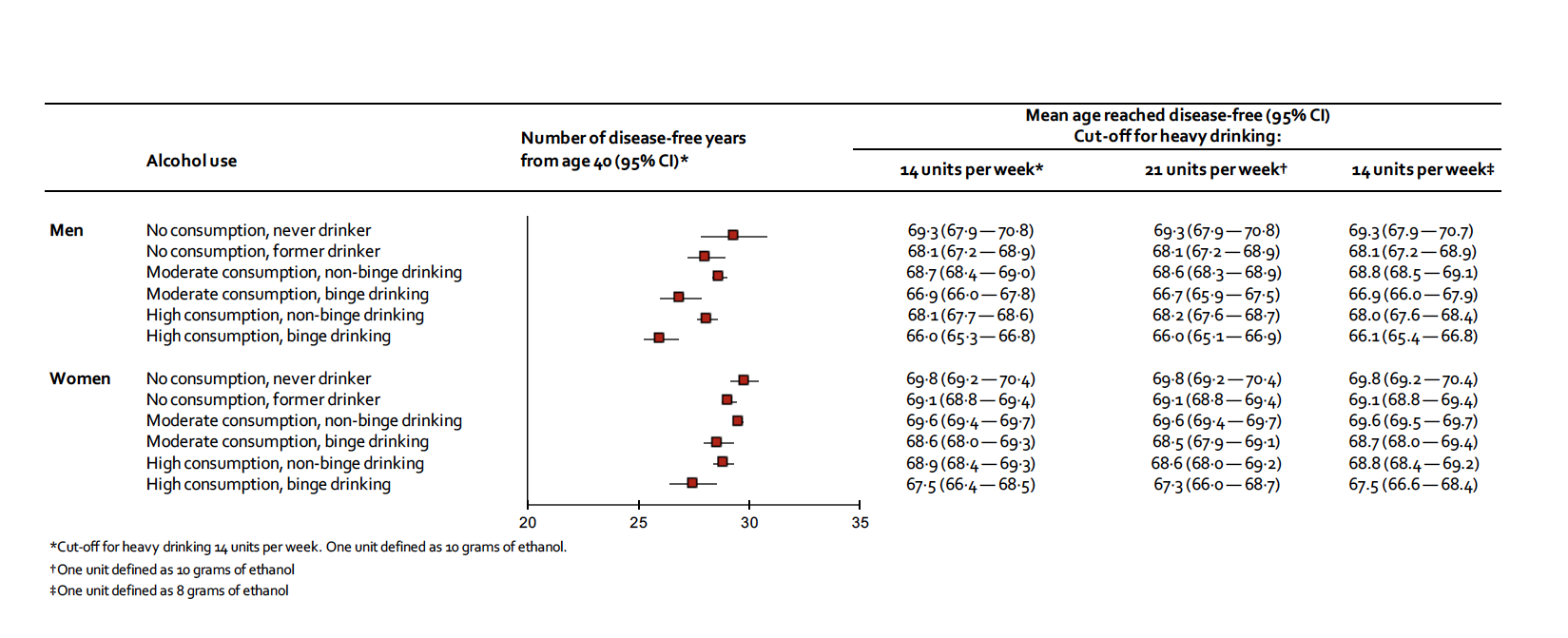


***Figure S1. Associations of alcohol consumption and binge drinking with disease-free years between ages 40 and 75 in the IPD-Work cohorts, using different thresholds for high consumption***


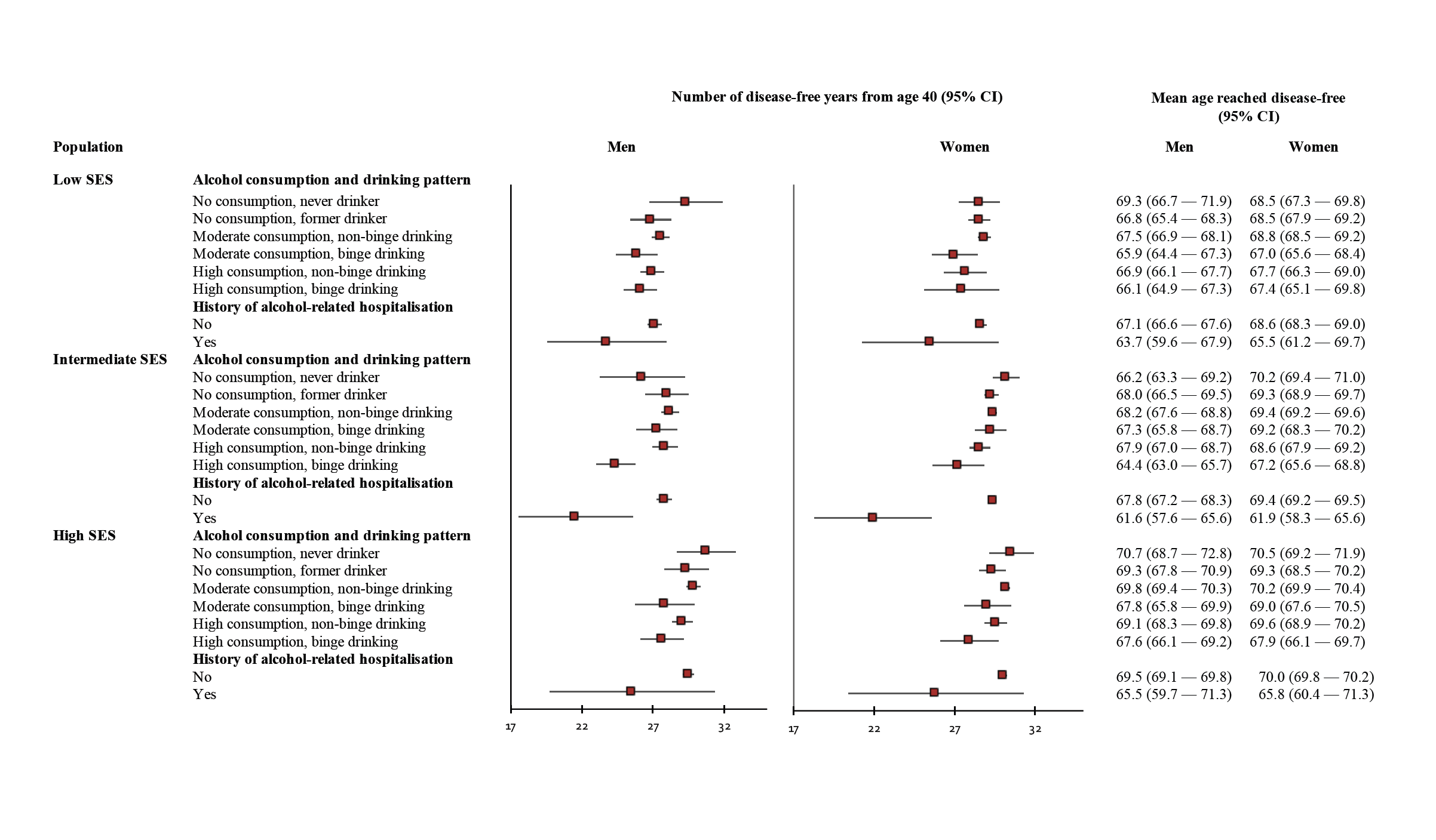


***Figure S2*. *Associations of alcohol consumption and binge drinking with disease-free years between ages 40 and 75 in the IPD-Work cohorts by socioeconomic status (SES)***


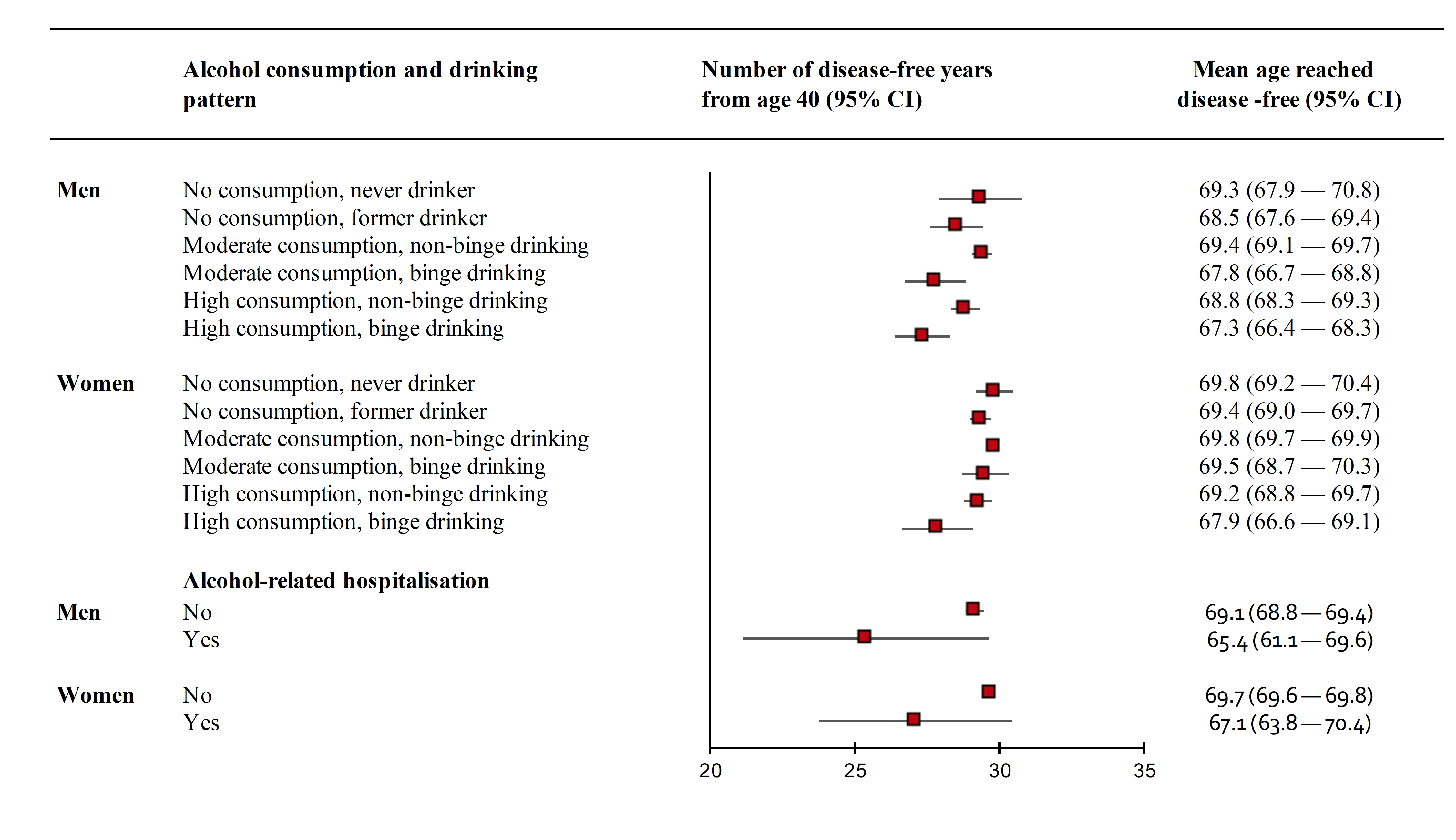


***Figure S3*. *Associations of alcohol consumption and binge drinking with disease-free years between ages 40 and 75 in the IPD-Work cohorts among non-smokers***


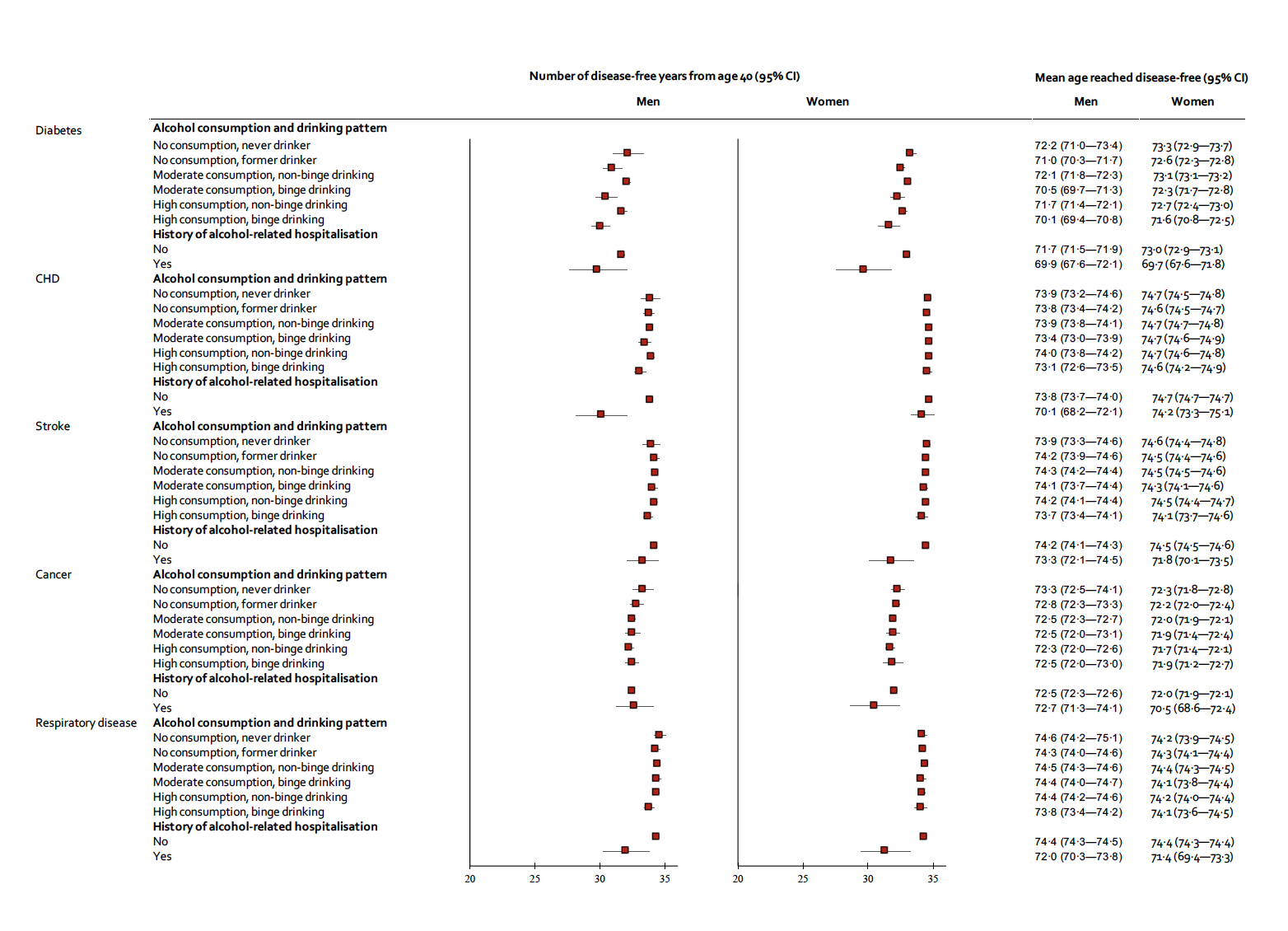


***Figure S4*. *Associations of alcohol consumption, binge drinking and alcohol-related hospitalisation with individual chronic conditions in the IPD-Work cohorts***


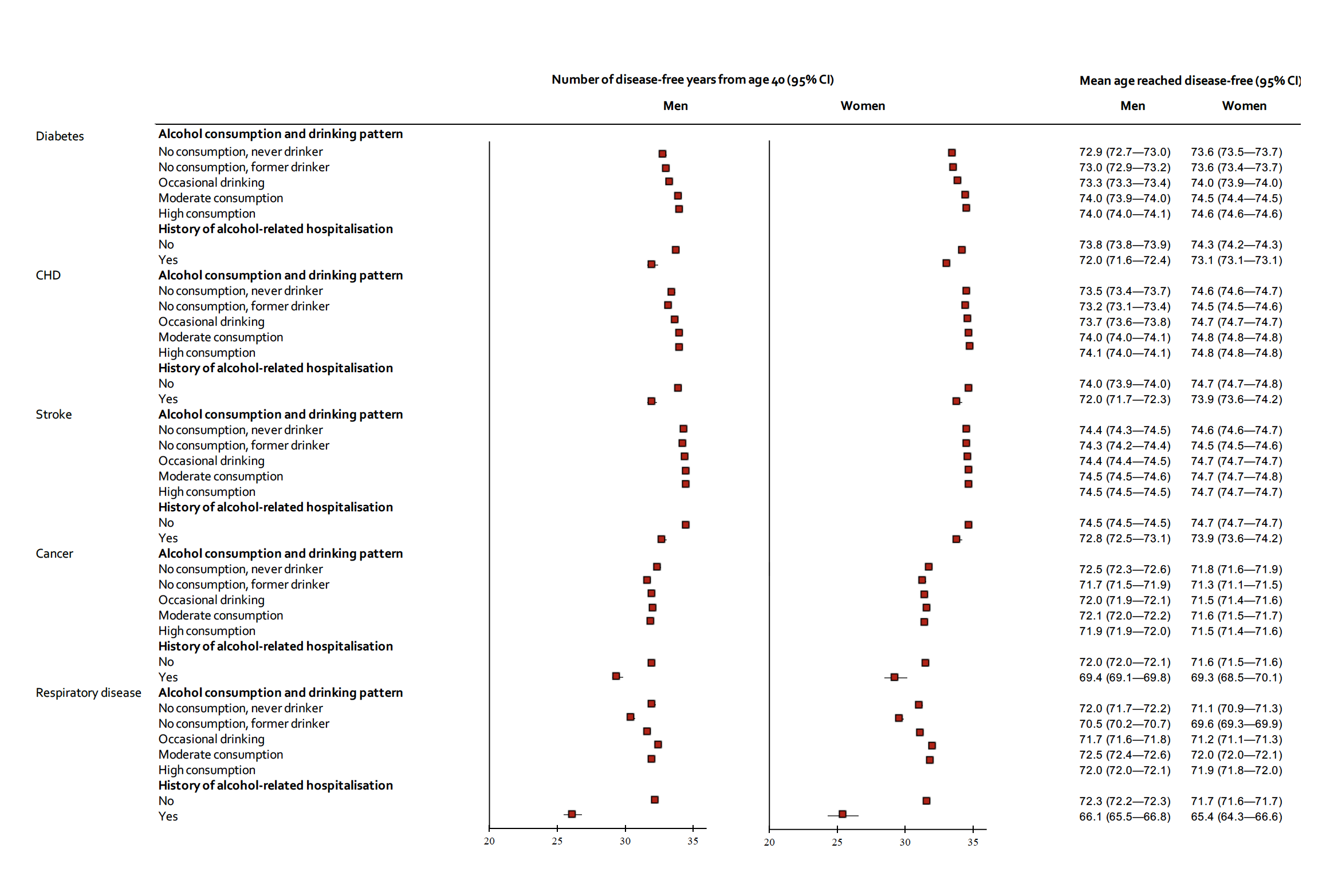


***Figure S5*. *Associations of alcohol consumption and alcohol-related hospitalisation with individual chronic conditions in UK Biobank***

**Statistical code**

**An example code for calculating the estimate disease-free life-years in the pooled dataset.**

foreach i of num 1/2 {

cd "somefolder"

insheet using data.txt, clear

drop if alkob214==.

gen group=1

replace group=2 if sex=="female"

tab group

drop id

gen id=_n

keep if group == `i'

gen alc=.

replace alc=0 if alkob214==3 //moderate, non-binge = reference

replace alc=1 if alkob214==1 //never

replace alc=2 if alkob214==2 //ex

replace alc=3 if alkob214==51 //moderate, binge

replace alc=4 if alkob214==4 //high non-binge

replace alc=5 if alkob214==52 //high, binge

stset loppupvm, failure(status_uh==1) id(id) origin(syntpvm) enter(basedate) exit(failure) scale(365.25)

egen t1 = seq(), f(4000) t(7500)

replace t1 = t1*0.01

summarize t1

foreach j in 1 2 3 4 5 {

capture noisily xi: stpm2 i.alc i.study, df(`j') scale(hazard) iterate(15)

local b1_`j'=el(r(table),2,1)

di `b1_`j''

gen AIC_df`j' = e(AIC) if e(converged)==1 & `b1_`j'' < .

}

capture noisily {

egen minni = rowmin(AIC_df*)

gen aikki=.

replace aikki=5 if AIC_df5==minni

replace aikki=4 if AIC_df4==minni

replace aikki=3 if AIC_df3==minni

replace aikki=2 if AIC_df2==minni

replace aikki=1 if AIC_df1==minni

di aikki

}

foreach k in 1 2 3 4 5 {

capture noisily xi: stpm2 i.alc i.study, df(`k') scale(hazard), if aikki==`k'

}

estat ic

mat b=e(b)'

predict S0, survival zero time(t1)

gen S0n = S0/S0[1]

gen S1 = S0n^exp(0)

gen S2 = S0n^exp(b[1,1])

gen S3 = S0n^exp(b[2,1])

gen S4 = S0n^exp(b[3,1])

gen S5 = S0n^exp(b[4,1])

gen S6 = S0n^exp(b[5,1])

integ S1 t1

gen iS1 = r(integral)

scalar LE_occ1 = iS1

integ S2 t1

gen iS2 = r(integral)

scalar LE_occ2 = iS2

integ S3 t1

gen iS3 = r(integral)

scalar LE_occ3 = iS3

integ S4 t1

gen iS4 = r(integral)

scalar LE_occ4 = iS4

integ S5 t1

gen iS5 = r(integral)

scalar LE_occ5 = iS5

integ S6 t1

gen iS6 = r(integral)

scalar LE_occ6 = iS6

drop S0 S1 S2 S3 S4 S5 S6 iS1 iS2 iS3 iS4 iS5 iS6 S0n

matrix YL = ( LE_occ1, LE_occ2, LE_occ3, LE_occ4, LE_occ5, LE_occ6)

capture program drop myboot

program define myboot, rclass

preserve

bsample

xi: stpm2 i.alc i.study, df(1) scale(hazard)

mat b=e(b)'

predict bS0, survival zero time(t1)

sort t1 bS0

gen bS0n = bS0/bS0[1]

gen bS1 = bS0n^exp(0)

gen bS2 = bS0n^exp(b[1,1])

gen bS3 = bS0n^exp(b[2,1])

gen bS4 = bS0n^exp(b[3,1])

gen bS5 = bS0n^exp(b[4,1])

gen bS6 = bS0n^exp(b[5,1])

integ bS1 t1

gen ibS1 = r(integral)

integ bS2 t1

gen ibS2 = r(integral)

integ bS3 t1

gen ibS3 = r(integral)

integ bS4 t1

gen ibS4 = r(integral)

integ bS5 t1

gen ibS5 = r(integral)

integ bS6 t1

gen ibS6 = r(integral)

return scalar YL_1 = ibS1

return scalar YL_2 = ibS2

return scalar YL_3 = ibS3

return scalar YL_4 = ibS4

return scalar YL_5 = ibS5

return scalar YL_6 = ibS6

drop bS0 bS1 bS2 bS3 bS4 bS5 bS6 ibS1 ibS2 ibS3 ibS4 ibS5 ibS6 bS0n

restore

end

simulate YL_1=r(YL_1) YL_2=r(YL_2) YL_3=r(YL_3) YL_4=r(YL_4) YL_5=r(YL_5) YL_6=r(YL_6) , reps(1000) seed(12345): myboot

bstat, stat(YL)

mat theta = e(b)

mat theta_se = e(se)

gen LE_1 = theta[1,1]

gen LE_2 = theta[1,2]

gen LE_3 = theta[1,3]

gen LE_4 = theta[1,4]

gen LE_5 = theta[1,5]

gen LE_6 = theta[1,6]

gen seLE_1 = theta_se[1,1]

gen seLE_2 = theta_se[1,2]

gen seLE_3 = theta_se[1,3]

gen seLE_4 = theta_se[1,4]

gen seLE_5 = theta_se[1,5]

gen seLE_6 = theta_se[1,6]

gen aikki=0

capture noisily {

keep aicsave LE_* seLE_*

save "/binge_LE_alc_`i'_`mmm'", replace

}

}

**An example code for summarizing cohort-specific results**

use "alc14_LE_ALL", clear

reshape long LE_ seLE_, i(group dg) j(alc14)

rename LE_ esti

rename seLE_ sele

egen dgsexstr = group(dg sex alc14), label

egen dgsex = group(dg sex alc14)

metan esti sele if (sex=="Women" & dg=="Any"), by(alc14) random label(namevar=study) lcols(study) t1title ("Any Women") xlabel(0, 20, 25, 30, 35) force nooverall nulloff

**References**

1. Pejtersen JH, Kristensen TS, Borg V, Bjorner JB. The second version of the Copenhagen Psychosocial Questionnaire. *Scandinavian journal of public health* 2010; **38**(3 Suppl): 8-24.

2. Burr H, Bjorner JB, Kristensen TS, Tüchsen F, Bach E. Trends in the Danish work environment in 1990–2000 and their associations with labor-force changes. *Scandinavian journal of work, environment & health* 2003; **29**(4): 270-9.

3. Feveile H, Olsen O, Burr H, Bach E. Danish Work Environment Cohort Study 2005: From idea to sampling design. *Statistics in Transition* 2007; **8**(3): 441-58.

4. Kivimäki M, Lawlor DA, Smith GD, et al. Socioeconomic Position, Co-Occurrence of Behavior-Related Risk Factors, and Coronary Heart Disease: the Finnish Public Sector Study. *American journal of public health* 2007; **97**(5): 874-9.

5. Goldberg M, Leclerc A, Bonenfant S, et al. Cohort profile: the GAZEL Cohort Study. *Int J Epidemiol* 2007; **36**(1): 32-9.

6. Korkeila K, Suominen S, Ahvenainen J, et al. Non-response and related factors in a nation-wide health survey. *Eur J Epidemiol* 2001; **17**(11): 991-9.

7. Lahelma E, Aittomaki A, Laaksonen M, et al. Cohort profile: the Helsinki Health Study. *Int J Epidemiol* 2013; **42**(3): 722-30.

8. Nielsen ML, Rugulies R, Christensen KB, Smith-Hansen L, Bjorner JB, Kristensen T. Impact of the psychosocial work environment on registered absence from work: a two-year longitudinal study using the IPAW cohort. *Work & Stress* 2004; **18**(4): 323-35.

9. Väänänen A, Murray M, Koskinen A, Vahtera J, Kouvonen A, Kivimäki M. Engagement in cultural activities and cause-specific mortality: prospective cohort study. *Preventive medicine* 2009; **49**(2-3): 142-7.

10. Marmot MG, Smith GD, Stansfeld S, et al. Health inequalities among British civil servants: the Whitehall II study. *Lancet* 1991; **337**(8754): 1387-93.

11. Alfredsson L, Hammar N, Fransson E, et al. Job strain and major risk factors for coronary heart disease among employed males and females in a Swedish study on work, lipids and fibrinogen. *Scandinavian journal of work, environment & health* 2002; **28**(4): 238-48.

12. Peter R, Alfredsson L, Hammar N, Siegrist J, Theorell T, P. W. High effort, low reward, and cardiovascular risk factors in employed Swedish men and women: baseline results from the WOLF Study. *Journal of epidemiology and community health* 1998; **52**: 540-7

13. . <https://www.ukbiobank.ac.uk/> (accessed February 3, 2022.

14. Townsend P, Phillimore P, Beattie A. Health and Deprivation: Inequality and the North: Croom Helm; 1988.

15. Disease GBD, Injury I, Prevalence C. Global, regional, and national incidence, prevalence, and years lived with disability for 354 diseases and injuries for 195 countries and territories, 1990-2017: a systematic analysis for the Global Burden of Disease Study 2017. *Lancet* 2018; **392**(10159): 1789-858.

16. Collaborators GBDCoD. Global, regional, and national age-sex-specific mortality for 282 causes of death in 195 countries and territories, 1980-2017: a systematic analysis for the Global Burden of Disease Study 2017. *Lancet* 2018; **392**(10159): 1736-88.

17. Kivimaki M, Hamer M, Batty GD, et al. Antidepressant medication use, weight gain, and risk of type 2 diabetes: a population-based study. *Diabetes Care* 2010; **33**(12): 2611-6.

18. Tabak AG, Jokela M, Akbaraly TN, Brunner EJ, Kivimaki M, Witte DR. Trajectories of glycaemia, insulin sensitivity, and insulin secretion before diagnosis of type 2 diabetes: an analysis from the Whitehall II study. *Lancet* 2009; **373**(9682): 2215-21.

19. Alberti KG, Zimmet PZ. Definition, diagnosis and classification of diabetes mellitus and its complications. Part 1: diagnosis and classification of diabetes mellitus provisional report of a WHO consultation. *Diabet Med* 1998; **15**(7): 539-53.

20. Kivimaki M, Nyberg ST, Batty GD, et al. Job strain as a risk factor for coronary heart disease: a collaborative meta-analysis of individual participant data. *Lancet* 2012; **380**(9852): 1491-7.

21. Fransson EI, Nyberg ST, Heikkila K, et al. Job strain and the risk of stroke: an individual-participant data meta-analysis. *Stroke* 2015; **46**(2): 557-9.

22. Heikkila K, Nyberg ST, Theorell T, et al. Work stress and risk of cancer: meta-analysis of 5700 incident cancer events in 116,000 European men and women. *BMJ (Clinical research ed* 2013; **346**: f165.

23. Heikkila K, Madsen IE, Nyberg ST, et al. Job strain and COPD exacerbations: an individual-participant meta-analysis. *Eur Respir J* 2014; **44**(1): 247-51.

24. Heikkila K, Madsen IE, Nyberg ST, et al. Job strain and the risk of severe asthma exacerbations: a meta-analysis of individual-participant data from 100 000 European men and women. *Allergy* 2014; **69**(6): 775-83.

25. Nyberg ST, Fransson EI, Heikkila K, et al. Job strain as a risk factor for type 2 diabetes: a pooled analysis of 124,808 men and women. *Diabetes Care* 2014; **37**(8): 2268-75.
