# Supplementary material for: Association of alcohol use with years lived without major chronic diseases: A multicohort study from the IPD-Work consortium and UK Biobank
Source: Lancet Reg Health Eur. 2022 May 29;19:100417. doi: 10.1016/j.lanepe.2022.100417 (PMC9160494; doi:10.1016/j.lanepe.2022.100417)
Supplement: Supplementary file 2 [file mmc2.docx]

Supplementary material
